# Protonation of Borylated Carboxonium Derivative [2,6-B_10_H_8_O_2_CCH_3_]^−^: Theoretical and Experimental Investigation

**DOI:** 10.3390/ijms23084190

**Published:** 2022-04-10

**Authors:** Ilya N. Klyukin, Anastasia V. Kolbunova, Alexander S. Novikov, Aleksey V. Nelyubin, Nikita A. Selivanov, Alexander Yu. Bykov, Alexandra A. Klyukina, Andrey P. Zhdanov, Konstantin Yu. Zhizhin, Nikolay T. Kuznetsov

**Affiliations:** 1 Kurnakov Institute of General and Inorganic Chemistry, Russian Academy of Sciences, Leninskii pr. 31, 117907 Moscow, Russia; zdimchi391@mail.ru (A.V.K.); nelyubin.av@yandex.ru (A.V.N.); goovee@yandex.ru (N.A.S.); bykov@igic.ras.ru (A.Y.B.); zhdanov@igic.ras.ru (A.P.Z.); zhizhin@igic.ras.ru (K.Y.Z.); boron@igic.ras.ru (N.T.K.); 2 Faculty of Chemistry, Higher School of Economics, Myasnitskaya yl., 101000 Moscow, Russia; 3 Institute of Chemistry, Saint Petersburg State University, Universitetskaya Nab. 7-9, 199034 Saint Petersburg, Russia; a.s.novikov@spbu.ru; 4 Research Centre of Biotechnology, Winogradsky Institute of Microbiology, Russian Academy of Sciences, 60 let Oktjabrja pr-t, 7, bld. 2, 117312 Moscow, Russia; alexandra.a.popova@gmail.com; 5 Lomonosov Institute of Fine Chemical Technologies, MIREA Russian Technological University, 78 Vernadskiy Avenue, 119571 Moscow, Russia

**Keywords:** *closo*-borates, boron cluster, protonation, DFT calculation, NMR spectra, QTAIM, Fukui function

## Abstract

The process of protonation of [2,6-B_10_H_8_O_2_CCH_3_]^−^ was investigated both theoretically and experimentally. The most suitable conditions for protonation of the derivative [2,6-B_10_H_8_O_2_CCH_3_]^−^ were found. The process of protonation was carried out in the presence of an excess of trifluoromethanesulfonic acid CF_3_SO_3_H at room temperature in dichloromethane solution. The structure of the resulting complex [2,6-B_10_H_8_O_2_CCH_3_*H^fac^]^0^ was established using NMR data and the results of DFT calculations. An additional proton atom H^fac^ was found to be localized on one of the facets that was opposite the boron atom in a substituted position, and which bonded mainly with one apical boron atom. The main descriptors of the B-H^fac^ bond were established theoretically using QTAIM and NBO approaches. In addition, the mechanism of [2,6-B_10_H_8_O_2_CCH_3_]^−^ protonation was investigated.

## 1. Introduction

The investigation of covalent and noncovalent interactions is one of the main tasks of modern inorganic chemistry [1,2]. Such studies provide an opportunity to better understand the structures of chemical substances and their properties. By examining chemical bonds, it is possible to establish factors that influence their breaking and formation [3,4]. This fact can be used to produce new substances with given properties. A combined theoretical and experimental approach is the best way to investigate such phenomena [5,6].

There are several driving forces behind bonding formation: orbital, electrostatic, and the van der Waals interactions [7,8,9]. Information about the energy characteristics of such interactions is one of the most important descriptors. Theoretical methods allow exploration of the nature of chemical interactions and estimation of their energies in a simple and intuitive way. The application of such popular and well-established methods as QTAIM (Quantum Theory of Atoms in Molecules), ELF (Electron Localization Function), and NBO (Natural Bond Orbitals) analysis provides the opportunity to find essential information about the chemical structure and bonding of target compounds [10,11,12,13,14].

*Closo*-borates and related compounds have always attracted attention due to their unusual structure. Their electron structures cannot be described in terms of the classic Lewis approach [11,15]. For a correct description of the structure of *closo*-borate compounds, the terms of 3D-aromacity are used. *Closo*-borates possess various interesting properties, such as thermodynamic and oxidative stability and biological activity [16,17,18]. These properties determine the potential application of *closo*-borate systems for the preparation of solid-state batteries [19], magnetic and hydrogen storage materials [20,21], and drugs for BNCT (boron neutron capture therapy) [22,23,24]. Such systems can form various covalent and noncovalent interactions. It is impossible not to mention the tremendous contributions of the pioneers of boron cluster chemistry, such as Lipscomb [25,26,27,28], Hoffman [29,30,31], Wade [32,33,34], Mingos [35,36,37], and Muetterties [38,39,40,41]. Their works have significantly expanded ideas about the structure and properties of such systems. Currently, there is a great deal of interest in the chemistry of *closo*-borate anions [42,43]. The phenomena of the *exo*-polyhedral B-X (X = H, C, N, O, F) bonds have been extensively studied [44,45,46], for example, research into the main characteristics of these bonds for hydroxy-derivatives [B_n_H_n-1_OH]^2−^ (*n* = 6, 10, 12) was carried out [47]. For the *exo*-polyhedral B-O bonds, it has been established that, for the *closo*-borate anion, covalent interactions increase with an increase in the boron cluster size. Noncovalent contacts of *closo*-borate anions and related compounds have also been investigated [48]. This type of interaction is weaker than covalent bonds but also has a significant effect on the structure and properties of the systems under study. For example, the interactions of nitrile derivatives with various nucleophiles have previously been studied [34,49,50,51,52]. It was shown that the structure of the products obtained was the result of noncovalent interactions between protons of nucleophile and hydrogen atoms of the cluster anion.

*Closo*-borate anions possess an excess of electron density and can act as an electron donor for an electrophilic system. They can form a complex with different Lewis acids: protons, AlCl_3,_ ML_n_, and others [42,53]. Previously, the structure of the [B_10_H_11_]^−^ anion was studied both theoretically and experimentally [54,55]. It was shown that H^fac^moves freely in the equatorial belt, with a very low energy barrier (approximately 1 kcal/mol) [20].

The present study focused on [2,6-B_10_H_8_O_2_CCH_3_]^−^. This anion is a member of the class of borylated heterocycles. Analogous derivatives based on borylated boroxazole were also investigated for *closo*-decaborate and dodecaborate anions [56,57,58,59]. Several approaches for the preparation of this class of *closo*-borate derivatives have been explored previously [60,61]. In addition, the disclosure reaction of borylated heterocycle has been established [62]. [2,6-B_10_H_8_O_2_CCH_3_]^−^ is a convenient model system for the investigation of the protonation process. This system is more symmetrical than mono-substituted derivatives and the number of possible isomers is reduced, compared to them. The protonation process of a given anion can be readily investigated. It is important to assess the differences and similarities between the protonation of the original anion [B_10_H_10_]^2−^ and the derivatives based on it. In addition, the protonation of a given derivative enables the preparation of a derivative with a degree of substitution equal to three. Thus, in the present research, a combined theoretical and experimental study of the protonation of [2,6-B_10_H_8_O_2_CCH_3_]^−^ was carried out.

## 2. Results and Discussion

### 2.1. Experimental Protonation

**Scheme 1:**
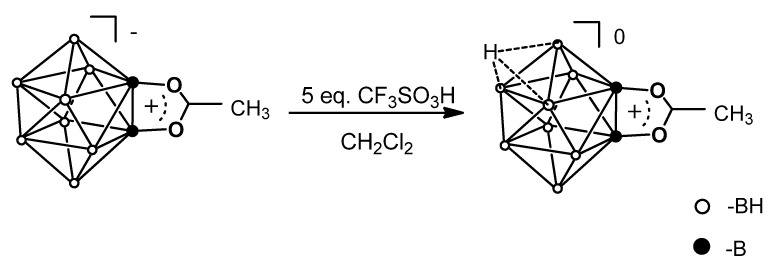
Protonation of carboxonium derivative [2,6-B_10_H_8_O_2_CCH_3_]^−^ anion.

First, the protonation process of [2,6-B_10_H_8_O_2_CCH_3_]^−^ was conducted experimentally. As described previously [63], systems of the general form [B_10_H_9_L]^−^ have a lesser possibility of forming a protonated complex than the [B_10_H_10_]^2−^ anion. A possible reason for this is that the introduction of a positively charged group reduces the ability of the cluster cage to donate its electron density. Anion [B_10_H_10_]^2−^ can be easily transferred into [B_10_H_11_]^−^ in the presence of trifluoroacetic acid CF_3_COOH. For the preparation of a protonated complex of the general form [B_10_H_9_OR_1_R_2_*H^fac^]^0^, however, trifluoromethanesulfonic acid CF_3_SO_3_H was used. In the present work, trifluoroacetic acid CF_3_COOH was also used as a proton donor, but the protonation process did not take place. As in the case of oxonium derivatives [B_10_H_9_OR_1_R_2_]^−^, the protonation of the derivative [2,6-B_10_H_8_O_2_CCH_3_]^−^ was carried out in the presence of CF_3_SO_3_H at room temperature (Scheme 1). Dichloromethane CH_2_Cl_2_ was used as a solvent. The application of another solvent was impossible due to the interaction of [2,6-B_10_H_8_O_2_CCH_3_*H^fac^]^0^ with the molecules of the solvents. [2,6-B_10_H_8_O_2_CCH_3_*H^fac^]^0^ was formed immediately after the addition of the acid. The distinctive feature of this process is that the protonation of the cluster anion requires an excess of CF_3_SO_3_H acid. For an investigation of these phenomena, the experiment with sequential addition of CF_3_SO_3_H acid to a dichloromethane solution of the initial [2,6-B_10_H_8_O_2_CCH_3_]^−^ derivative was conducted. The process was monitored by ^11^B-NMR spectroscopy (Figure S1). Detailed information about the NMR spectra of the initial and target substances and the correlation of all signals are given below in the NMR spectra analysis section. When one equivalent of acid was added to the system, the overall structure of the spectrum characteristic of the original derivative was preserved, but one of the signals was broadened. This signal corresponds to boron atoms in apical positions. The given pattern indicates the possibility of the formation of intermolecular contacts between the carboxonium derivative and the trifluoromethanesulfonic acid molecule. When one more acid equivalent is added to the system, a more significant change in the spectrum was observed, which may indicate the formation of molecular complexes between the [2,6-B_10_H_8_O_2_CCH_3_]^−^ derivative and the acid molecule. The formation of the target substance was also observed, as indicated by the presence of a broadened signal in the region of 20 ppm. However, the yield of the target product did not exceed approximately 10 percent. A more accurate estimation of the target product yield could not be made due to the overlapping signals of the target product and the cluster-acid complex. At ratios [2,6-B_10_H_8_O_2_CCH_3_]^−^:CF_3_SO_3_H equal to 1:3 and 1:4, an increase in the yield of the target product was observed. In the case of the 1:4 ratio, the yield of the target product reached approximately 40 percent. Finally, in the presence of 5 equivalents of CF_3_SO_3_H, complete protonation of [2,6-B_10_H_8_O_2_CCH_3_]^−^ with formation of [2,6-B_10_H_8_O2CCH_3_*H^fac^]^0^ occurred. Further addition of the acid excess did not change the form of the spectrum, indicating the completeness of the protonation process. It is noteworthy that [2,6-B_10_H_8_O_2_CCH_3_*H^fac^]^0^ is stable only as a solvent at room temperature and without air.

### 2.2. Fukui Function

**Figure 1:**
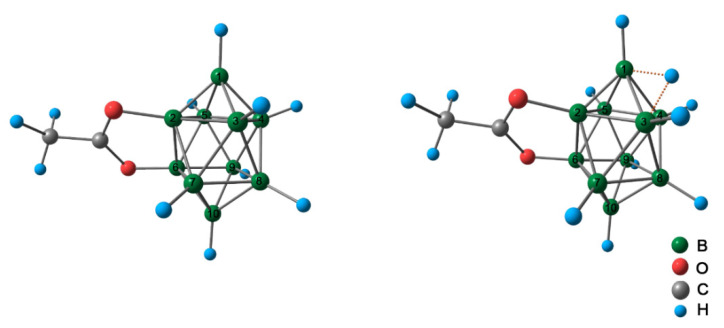
Optimized structures of [2,6-B_10_H_8_O_2_CCH_3_]^−^ and [2,6-B_10_H_8_O_2_CCH_3_*H^fac^]^0^.

In the next step, the potential position in the boron cluster for the coordination of an additional proton atom was investigated. The protonation process of [2,6-B_10_H_8_O_2_CCH_3_]^−^ can be considered to be an electrophilic attack on the boron cluster. The Fukui functions approach is the best tool for investigation of the most likely position in the molecule for an electrophilic or nucleophilic attack [64,65,66]. Previously, it has been shown, with the help of Fukui functions, that electrophilic attack on closo-borate anions is performed predominantly in the apical position [67,68]. In the present investigation, Fukui functions were used with different approaches employed for their calculation (Table S2). As in the case of [B_n_H_n_]^2−^ systems [68], the Hirshfeld approach is the best for calculating the electrophilic attack positions. In the present case, electrophilic attack on closo-borate anions was performed predominantly in the apical position (the optimized structures of [2,6-B_10_H_8_O_2_CCH_3_]^−^ and [2,6-B_10_H_8_O_2_CCH_3_*H^fac^]^0^ are shown in Figure 1). Thus, the data from the Fukui functions analysis indicated that the H^fac^ was localized near apical positions (Table S3).

### 2.3. Protonation Mechanism

**Figure 2:**
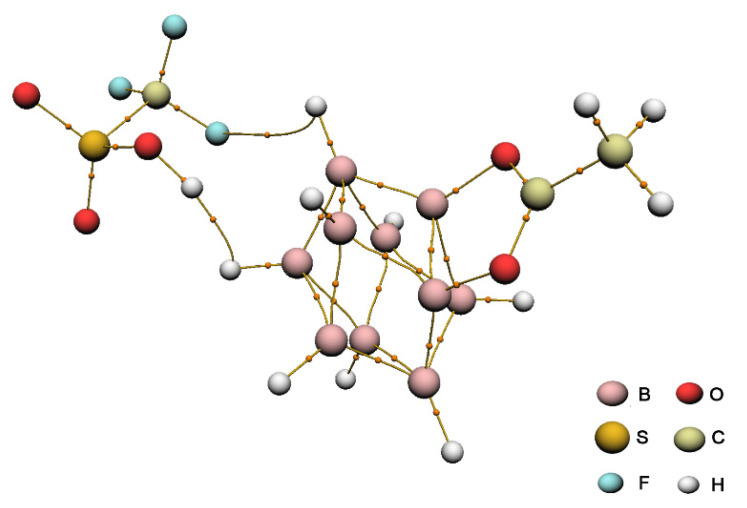
Molecular graph showing the results of the topological analysis of the electron density distribution in the model structure of the [2,6-B_10_H_8_O_2_CCH_3_^*^CF_3_SO_3_H]^−^ (Comp).

The mechanism of [2,6-B_10_H_8_O_2_CCH_3_]^−^ protonation was investigated. Initially, complex [2,6-B_10_H_8_O_2_CCH_3_^*^CF_3_SO_3_H]^−^ (Comp) formed endoergonically (by 23 kJ·mol^−1^, in terms of Gibbs free energies). The main driving force behind the formation of this complex is the dihydrogen bond between the proton atom from CF_3_SO_3_H and the equatorial hydrogen atom from the boron polyhedron. The contact length of H_eq_–H was equal to 1.60 Å. The presence of the dihydrogen bond was proved with the help of QTAIM analysis of the Comp structure (Figure 2). In addition, the C–F⋯H–B contact was detected. According to the main topological descriptors of electron density C–F⋯H–B, contact can be characterized as being very weak.

**Figure 3:**
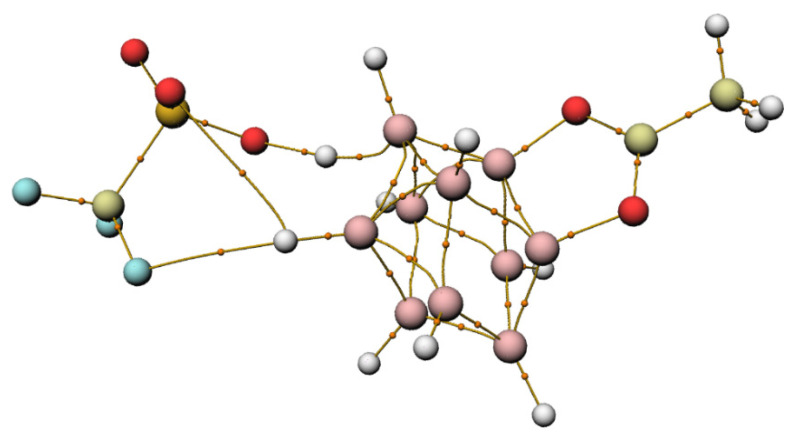
Molecular graph showing the results of the topological analysis of the electron density distribution in the model structure of TS. Atomic designations are given in Figure 2.

In the next step, the proton migrated to a boron polyhedral through the formation of the transition state (TS). The preferred location for the proton attack was the face of the polyhedron opposite the substituted position. The proton from CF_3_SO_3_H acid in TS connected mainly with the boron atom in the apical position, which was demonstrated with the help of QTAIM analysis (Figure 3). In the case of TS, the contact length between the apical boron atom and the proton atom from CF_3_SO_3_H was equal to 1.48 Å. The overall energy barrier of proton migration was 69 kJ·mol^−1^.

**Figure 4:**
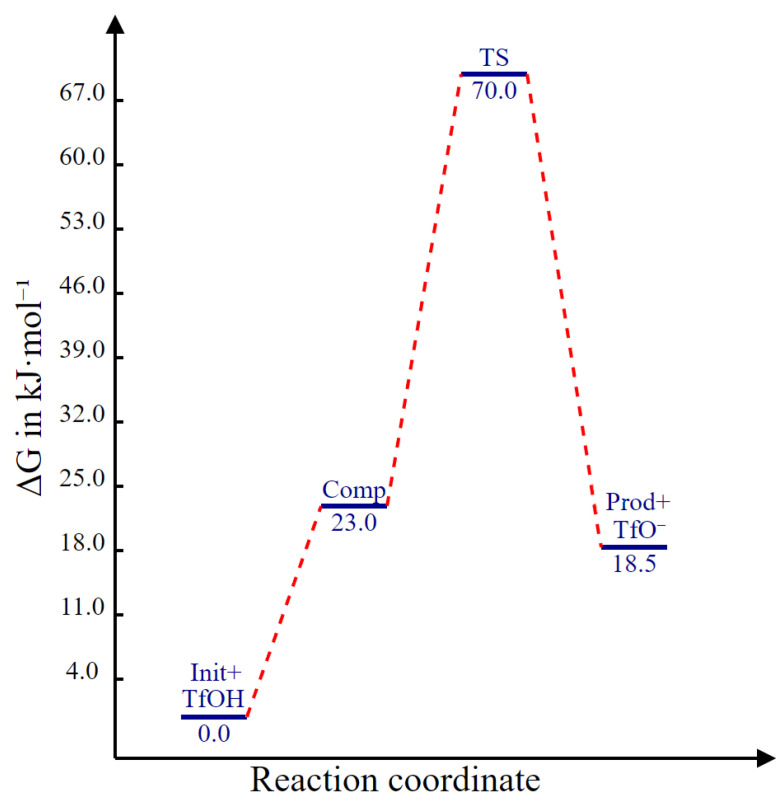
Energy profile of protonation of [2,6-B_10_H_8_O_2_CCH_3_]^−^. TfOH–CF_3_SO_3_H, TfO^−^–CF_3_SO_3_^−^. The deciphering of other abbreviations is given in the main text.

Finally, [B_10_H_8_O_2_CCH_3_*H^fac^]^0^ formed. The overall process of boron cluster protonation is endoergonical (by 18.5 kJ·mol^−1^, in terms of Gibbs free energies) (Figure 4). Thus, due to ΔG > 0 overall, a reaction did not occur in the presence of 1 equivalent of CF_3_SO_3_H. This finding correlates well with experimental data and the main reason it occurred is the weak stabilization of CF_3_SO_3_^−^ in the dichloromethane solution. Additional proton donors are required to stabilize this anion. The use of an excess of trifluoromethanesulfonic acid can provide this. The CF_3_SO_3_^−^ can be stabilized by hydrogen bonds between oxygen atoms of this anion and hydrogen atoms of CF_3_SO_3_H. For example, complex between CF_3_SO_3_^−^ anion and CF_3_SO_3_H was observed (Figure S8). This complex possesses strong hydrogen bonds and the formation of a given system is an exergonic process. It is worth noting that the addition of the acid excess also significantly changed the properties of the medium, which in turn can change the solvation energy of the model system under consideration. This aspect may also contribute to the fact that the protonation process becomes exergonic when an excess of the acid is supplied.

### 2.4. NMR Spectra Analysis

**Figure 5:**
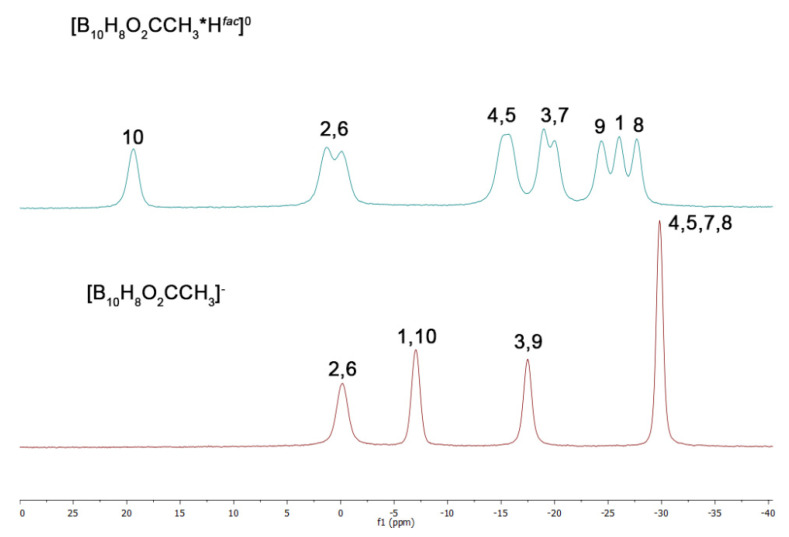
^11^B-^1^H NMR spectra of [B_10_H_8_O_2_CCH_3_]^−^ (**bottom**) and [B_10_H_8_O_2_CCH_3_*H^fac^]^0^ (**top**). The numbers are the positions of the boron atoms in the cluster polyhedral.

^11^B NMR spectra were used for the confirmation suggestion based on the Fukui index results and to establish the structure of the resulting complex. It is useful to consider the spectra of the [2,6-B_10_H_8_O_2_CCH_3_]^−^ anion. Due to the high symmetry of the given system, the two boron atoms in the substituted positions and the two apical atoms are pairwise equivalent. Atoms in equatorial positions are split into two groups of signals (Figure 5). Thus, as described previously, the ^11^B NMR spectra of the initial anion were characterized by four signals, as follows: a signal at 0.0 ppm with integral intensities I = 2, corresponding to the boron atoms from equivalent substituted positions. This signal did not split in the absence of broadband decoupling; a signal at −7.1 with integral intensities I = 2, corresponding to boron atoms from equivalent apical positions; and signals from equatorial positions appeared at −17.6 (I = 2) and −30.0 (I = 4). This interpretation of the spectrum is based on previously obtained spectral data for several *closo*-decaborate derivatives with B-O *exo*-polyhedral bonds. This approach does not, however, enable a complete correlation of signals and leaves some room for speculation.

The theoretical calculation of chemical shielding enabled the full correlation of the signals in the ^11^B NMR spectra with the boron positions in a cluster cage. Several computational levels for calculating NMR shifts were tested: wB97X-D3, B3LYP, and B97 DFT-functionals with 6–31++G(d,p), IGLO-III, and EPR-III basis sets (Table S5). To evaluate the difference between the theoretical and experimental data, the values of the root-mean-square deviation (RMSD) were used. The wB97X-D3 level of theory provided the worst results, with all basis sets. The values of RMSD were quite large (the range of RMSD values lay in the range 46–47) and applying this method did not enable the deciphering of the ^11^B spectra of given compounds. Improved results were obtained with the application of the hybrid functionals B3LYP and B97. In the case of the B97 functional, the best values of RMSD were obtained (Table S5). The RMSD value for the B97/IGLO-III level of theory was equal to 3.7. Thus, applying the results of the theoretical calculation, the assumption about the chemical shifts of atoms in the substituted and apical positions proved to be correct. In addition, the signal at −17.6 ppm corresponded to boron atoms from the equatorial belt in the B3 and B9 positions. The signal from −30.0 ppm corresponded to atoms in the B4, B5, B7, and B8 positions.

The ^1^H NMR spectra of [2,6-B_10_H_8_O_2_CCH_3_]^−^ were considered. For a simple view of the spectrum and the correct correlation of signals, it is best to use ^1^H-^11^B NMR spectra (Figure S2). Some of the signals appeared, however, as broadened lines, making it difficult to identify them. Signals from the methyl substituent appeared at 2.18 ppm, and signals from apical positions at 3.16 ppm were well represented. Signals from equatorial positions appeared at 1.79, 0.39 and 0.12 ppm.

The addition of a proton reduced the symmetry of the resulting system and the spectra of [2,6-B_10_H_8_O2CCH_3_*H^fac^]^0^ were more complicated than those of [2,6-B_10_H_8_O_2_CCH_3_]^−^. Boron atoms were split into separate signals. Thus, it was impossible to elucidate the structure of [2,6-B_10_H_8_O2CCH_3_*H^fac^]^0^ by only applying the 1D ^11^B spectra. To decipher these spectra, the ^11^B-^11^B COSY spectra and theoretical calculations were used (Figure S5). This type of spectrum indicates that, contrary to the case of the [B_10_H_11_]^−^ anion, the proton in this anion does not migrate along one of the equatorial belts but is localized on one of the edges. Based on the calculated data, the most stable isomer Iso1 was found. In this isomer, the proton was localized on the edge opposite the substituted position. The structure of this isomer is described in more detail below. Similar to the initial anion [2,6-B_10_H_8_O_2_CCH_3_]^−^, the B97 functional worked well for the prediction of NMR spectra. The RMSD value for the B97/IGLO-III level of theory was equal to 3.5 (Table S5).

As in the case of [B_10_H_11_]^−^, the anion [2,6-B_10_H_8_O_2_CCH_3_*H^fac^]^0^ has two non-equivalent apical positions. The first of them is bonded with additional proton atoms H^fac^ and the second one is not. Signal from the boron atom of the apical position B10, which was not bonded with H^fac^, shifted into a low field and appeared at 19.4 ppm. Similar observations were made in the case of the apical position B10 of the [B_10_H_11_]^−^ anion. In addition, the existence of this shift was proved by theoretical calculation. According to theoretical calculation, the signal of the apical position B10 appeared at 13.4 ppm. The chemical shift from the apical position B1, which bonded with H^fac^, shifted in the high field and appeared at −26.9 ppm. The boron atoms in the substituted positions were also not equivalent in the case of a protonated cluster. The signal at 0.2 ppm correlated with the B10 position of the boron cluster in the ^11^B-^11^B COSY NMR spectra, thus it corresponded to the B6-substituted position, which is opposite to the apical position with H^fac^. Another substituted position was found at −1.6 ppm. At −15.4 ppm, the signal from the B4,5 positions appeared. Signals from the B3 and B7 positions were relatively close, appearing at −19.0 and −20.1 ppm, respectively. Signals from the B9 and B8 positions had the greatest negative chemical shift value at −26.1 and −27.8 ppm, respectively.

In the ^1^H NMR spectra of [2,6-B_10_H_8_O_2_CCH_3_*H^fac^]^0^, the signal of the methyl group was observed at 2.45 ppm (Figure S3). Signals from the exo-polyhedral cluster were in the range 5.16–0.33 ppm. It is noteworthy that the signal at 5.16 ppm corresponded to a hydrogen atom connected with a boron atom in the B10 position. This assumption is based on the result of ^1^H NMR spectra modeling. According to the results of the calculations, the proton in the downfield region belonged to the given atom. The signal from H^fac^_,_ according to theoretical calculations, was in the downfield region at 0.00 ppm. In the experimental spectra, the most upfield signal was at 0.33 ppm. In the ^13^C NMR spectra of [2,6-B_10_H_8_O_2_CCH_3_*H^fac^]^0^, the signal of the carbonyl group carbon atom was observed at 194.0 ppm and the signal of the methyl group carbon atom was observed at 20.10 ppm (Figure S4). In the IR-spectra, the B-H bands were shifted from 2490 cm^−1^ in the initial [2,6-B_10_H_8_O_2_CCH_3_]^−^ to 2540 cm^−1^ in [2,6-B_10_H_8_O_2_CCH_3_*H^fac^]^0^ (Figure S6).

### 2.5. Structure Elucidation

**Figure 6:**
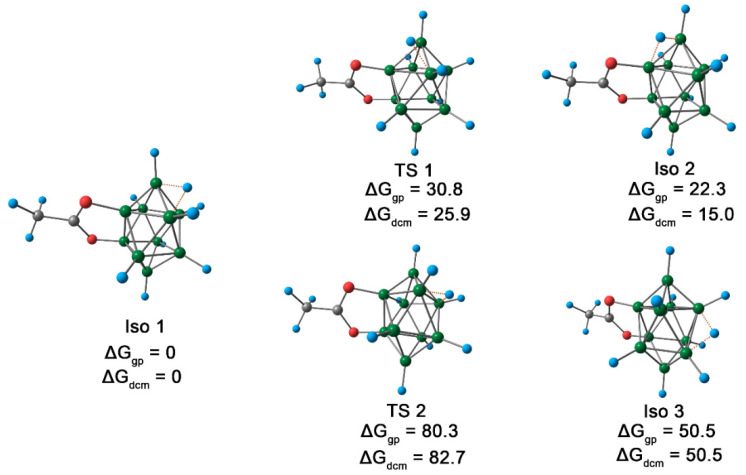
Optimized structures of the main isomers of [2,6-B_10_H_8_O_2_CCH_3_*H^fac^]^0^. The atomic designations are given in Figure 1. ΔG_gp_—relative Gibbs energy of isomer in gas phase. ΔG_dcm_—relative Gibbs energy of isomer in dichlormethane solution.

The structure of [2,6-B_10_H_8_O_2_CCH_3_*H^fac^]^0^ was examined using DFT calculation. Several possible isomers were calculated (Figure 6 and Figure S7, Table S4). Calculations were performed in the gas phase and considering the solvation effect. In both cases, the isomers differed significantly in terms of energy. The Iso1 and Iso2 isomers were the most thermodynamically stable (Figure 6). In these isomers, an extra proton, in addition to the equatorial boron atoms, was localized on the apical boron atom. The Iso1 in which the proton was localized on the edge opposite the substituted position had the highest negative Gibbs energy among all isomers. The energetic barrier for the migration of the proton atom from Iso1 to Iso2 was equal to 30 kJ·mol^−1^in the gas phase and 25 kJ·mol^−1^ in dichloromethane solution. Isomers with the localized proton only on equatorial boron atoms were the least thermodynamically stable and had the smallest negative Gibbs energy values. The energy barrier between Iso1 and Iso3 was 50 kJ·mol^−1^ for both the gas and dichloromethane phase. This energy barrier was less than that of the anion [B_10_H_11_]^−^. Thus, Iso1 was the most stable isomer among the other possible structures of [2,6-B_10_H_8_O_2_CCH_3_*H^fac^]^0^.

**Figure 7:**
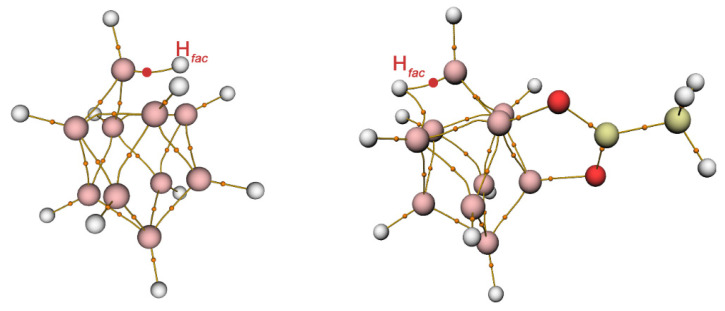
Molecular graph showing the results of the topological analysis of the electron density distribution in the model structures of [B_10_H_11_]^−^ and [2,6-B_10_H_8_O_2_CCH_3_*H^fac^]^0^. The atomic designations are given in Figure 2.

The geometric parameters of this isomer were discussed in detail (Table S1). The bond length between H^fac^ and the apical boron atom was 1.28 Å, whereas the analogous parameter for the bond between H^fac^ and the equatorial boron atom was 1.42 Å. The bond length between equatorial boron atoms and the apical boron atom that bonded with the proton increased compared to the same parameter in the original cluster. This parameter increased from 1.72 to 1.84 Å. The phenomena of B-H^fac^ contacts were investigated using Wiberg bond order indices and QTAIM analysis (Figure 7). Applying the QTAIM method, a molecular graph of the electron density distribution in [2,6-B_10_H_8_O_2_CCH_3_*H^fac^]^0^ was obtained. The molecular graph indicated that H^fac^ has interactions with apical and equatorial boron atoms. The B_ap_–H^fac^ interaction was characterized by a greater value of the Wiberg bond order index compared to that of B_eq_–H^fac^. The B_ap_–H^fac^ interaction was also characterized by greater values of ρ(*r*), total energy at the bond critical point, and the delocalization index. These data indicate that H^fac^ is predominantly bonded with the boron atom in the apical position. An analogous investigation was carried out for the case of the [B_10_H_11_]^−^ anion. For this anion, H^fac^ only bonded with the boron atom in the apical position. This contact was characterized by a longer B_ap_-H^fac^ bond and smaller values of the main bond descriptors. Thus, in the case of [2,6-B_10_H_8_O_2_CCH_3_*H^fac^]^0^ B_ap_–H^fac^ has greater covalent interaction than the [B_10_H_11_]^−^ anion.

The values of the main bond descriptors indicated that the B_ap_–H^fac^ interactions were significantly weaker than those of the covalent interactions between boron atoms and exo-polyhedral hydrogen atoms. It is noteworthy that the values of the main bond descriptors of B-H and B-O bonds were greater in the case of [2,6-B_10_H_8_O_2_CCH_3_*H^fac^]^0^ than in [2,6-B_10_H_8_O_2_CCH_3_]^−^. This indicates that the strength of these contacts increases. A possible reason for these phenomena may be the reduction in the total charge in the system compared to that of the initial anion [2,6-B_10_H_8_O_2_CCH_3_]^−^. The atomic charges on the boron atoms are reduced and the electron repulsion in the exo-polyhedral bonds is also reduced.

## 3. Materials and Methods

### 3.1. Computational Details

The full geometry optimization of all model structures was carried out at the ωB97X-D3/6–31++G(d,p) level of theory [69,70] with the help of the ORCA 4.2.1 program package (Mülheim an der Ruhr, Germany) [71] (the atom-pairwise dispersion correction with the zero-damping scheme was utilized [70]). The convergence tolerances for the geometry optimization procedure were as follows: energy change = 5.0 × 10^−6^ Eh, maximal gradient = 3.0 × 10^−4^ Eh/Bohr, RMS gradient = 1.0 × 10^−4^ Eh/Bohr, maximal displacement = 4.0 × 10^−3^ Bohr, and RMS displacement = 2.0 × 10^−3^ Bohr. Spin-restricted approximation for the model structures with closed electron shells was used. Symmetry operations were not applied during the geometry optimization procedure for any of the model structures. The Hessian matrices were calculated numerically for all optimized model structures in order to prove the location of correct minima on the potential energy surfaces (no imaginary frequencies for all reactants, intermediates, and final products; only one imaginary frequency for transition states). The connectivity of each reaction step was also confirmed using the intrinsic reaction coordinate (IRC) calculation from the transition states [72,73,74]. Solvent effects were considered using the Solvation Model based on Density (SMD) [75]. The natural bond orbital (NBO) method was emplyed, using the NBO7 program package (Madison, WI, USA) [76]. Topological analysis of the electron density distribution, using the Quantum Theory of Atoms in Molecules (QTAIM) formalism developed by Bader [12], was employed with the Multiwfn program, version 3.7 (Beijing, China) [77]. The Cartesian atomic coordinates for all optimized equilibrium model structures are presented in the Supplementary Materials. Visualization of the optimized structures was carried out with the help of ChemCraft program version 1.7 (Ivanovo, Russia) [78]. In the case of the molecular graph showing the results of the topological analysis of the electron density distribution visualization, the Multiwfn program (version 3.7) was employed [77].

### 3.2. IR Spectra

The IR spectra of the prepared compounds were recorded on an Infralyum FT 02 Fourier transform spectrometer (Lumex Instruments Research and Production Company, Fraserview Place, Vancouver, BC, Canada) in the region of 300–4000 cm^–1^ and with a resolution of 1 cm^–1^. Samples were prepared as dichloromethane CH_2_Cl_2_ solution.

### 3.3. NMR Spectra

The NMR (^1^H, ^11^B, ^13^C) spectra of the solutions of the studied compounds in CD3CN were recorded on a Bruker Avance II 300 spectrometer (Ettlingen, Germany) operating at 300.3, 96.32, and 75.49 MHz, respectively, using an internal deuterium lock. Tetramethylsilane and boron trifluoride etherate were used as external references.

### 3.4. Protonation of ((C_6_H_5_)_4_P)[2,6-B_10_H_8_O_2_CCH_3_]

CF_3_SO_3_OH (0.018 mL, 1.5 mmol) was added to a solution of ((C_6_H_5_)_4_P)[2,6-B_10_H_8_O_2_CCH_3_] (20 mg, 0.3 mmol) in deuterated dichloromethane CD_2_Cl_2_ (0.5 mL), in a dry argon atmosphere. The resulting mixture was kept at room temperature for 10 min. IR-spectra (dichloromethane solution): ʋ(BH) 2590 cm^−1^, 2555 cm^−1^. ^11^B-{^1^H} NMR (CD_2_Cl_2_, ppm): 19.2 (s, B10, I = 1), 1.4 (d, B6, I = 1), −0.2 (d, B2, I = 1), −15.2 (d, B4, I = 1), −15.7 (d, B3, I = 1), −19.1 (d, B5, I = 1), −20.1 (d, B9, I = 1), −24.4 (d, B7, I = 1), −26.1 (d, B1, I = 1), −27.8 (d, B8, I = 1). 1H NMR (CD_2_Cl_2_, ppm): 11.75 (CF_3_SO_3_H), 7.97 (m, Ph_4_P^+^, I = 4), 7.80 (m, Ph_4_P^+^, I = 8), 7.66 (m, Ph_4_P^+^, I = 8), 5.17 (s, B(10)H, I = 1), 2.68 (s, B(3)H, I = 1), 2.50 (s, CH_3_, I = 3), 2.03 (s, B(9)H, I = 1), 1.60 (s, B(4)H, I = 1), 1.32 (s, B(5)H, I = 1), 0.93 (s, B(7)H, I = 1), 0.62 (s, B(8)H, I = 1), 0.36 (s, H^fac^, I = 1). ^13^C NMR (CD_2_Cl_2_, ppm): 194.7 (O2CCH3), 140.6 (Ph_4_P+), 139.8 (Ph_4_P_+_), 135.8 (Ph_4_P^+^), 122.7 (Ph_4_P^+^), 118.1 (CF_3_SO_3_H), 20.1 (O_2_CCH_3_).

## 4. Conclusions

The process of protonation of the carboxonium derivative [2,6-B_10_H_8_O_2_CCH_3_]^−^ was investigated. In the case of the carboxonium derivatives of *closo*-decaborate anions, trifluoromethanesulfonic acid CF_3_SO_3_H was used as a proton donor. By considering the reaction mechanisms, the reason for the excess of CF_3_SO_3_H was established. The excess acid was required to stabilize the anion CF_3_SO_3_^−^, which in turn caused the total protonation process to become exergonic. In contrast to the anion [B_10_H_11_]^−^, in the case of [2,6-B_10_H_8_O_2_CCH_3_*H^fac^]^0^, the proton did not migrate along the equatorial belt and was localized on a facet opposite the B atoms bonded with the exo-polyhedral substituent. Based on theoretical modeling data, it was shown that the proton was predominantly bound to the apical boron atom. In addition, the ^11^B-^1^H NMR spectra of the final compound were quite complicated and difficult to decipher. Using theoretical modeling data, all signals were correlated with the positions of the boron atoms in the cluster. Theoretical calculations at the B97/IGLO-III level of theory corresponded perfectly with the experimental ^11^B-^1^H NMR spectra data. [2,6-B_10_H_8_O_2_CCH_3_*H^fac^]^0^ can be considered to be a promising synthon for the preparation of trisubstituted derivatives of closo-borate anion.

## Data Availability

Not applicable.

## Supplementary Materials

The following supporting information can be downloaded at: https://www.mdpi.com/article/10.3390/ijms23084190/s1.

## References

[1] Pino-Rios R., Inostroza D., Tiznado W. (2021). Neither too Classic nor too Exotic: One-Electron Na⋅B Bond in NaBH ^3^^−^ Cluster. Angew. Chem. Int. Ed..

[2] Su B., Cao Z.-C., Shi Z.-J. (2015). Exploration of Earth-Abundant Transition Metals (Fe, Co, and Ni) as Catalysts in Unreactive Chemical Bond Activations. Acc. Chem. Res..

[3] Woodward R.B., Hoffmann R. (1969). The Conservation of Orbital Symmetry. Angew. Chem. Int. Ed..

[4] Abu-Youssef M.A.M., Langer V., Barakat A., Haukka M., Soliman S.M. (2021). Molecular, Supramolecular Structures Combined with Hirshfeld and DFT Studies of Centrosymmetric M(II)-azido {M = Ni(II), Fe(II) or Zn(II)} Complexes of 4-Benzoylpyridine. Symmetry.

[5] Gouda M., Ferjani H., El-Lateef H.M.A., Khalaf M.M., Shaaban S., Yousef T.A. (2022). A Competition between Hydrogen, Stacking, and Halogen Bonding in *N*-(4-((3-Methyl-1,4-dioxo-1,4-dihydronaphthalen-2-yl)selanyl)phenyl)acetamide: Structure, Hirshfeld Surface Analysis, 3D Energy Framework Approach, and DFT Calculation. Int. J. Mol. Sci..

[6] Kolomeychuk F.M., Safonova E.A., Polovkova M.A., Sinelshchikova A.A., Martynov A.G., Shokurov A.V., Kirakosyan G.A., Efimov N.N., Tsivadze A.Y., Gorbunova Y.G. (2021). Switchable Aromaticity of Phthalocyanine via Reversible Nucleophilic Aromatic Addition to an Electron-Deficient Phosphorus(V) Complex. J. Am. Chem. Soc..

[7] Weinhold F., Landis C.R. (2012). Discovering Chemistry with Natural Bond Orbitals.

[8] Lepetit C., Fau P., Fajerwerg K., Kahn M.L., Silvi B. (2017). Topological analysis of the metal-metal bond: A tutorial review. Coord. Chem. Rev..

[9] Cortes-Guzman F., Bader R. (2005). Complementarity of QTAIM and MO theory in the study of bonding in donor-acceptor complexes. Coord. Chem. Rev..

[10] Kumar P.S.V., Raghavendra V., Subramanian V. (2016). Bader’s Theory of Atoms in Molecules (AIM) and its Applications to Chemical Bonding. J. Chem. Sci..

[11] Bader R.F.W., Legare D.A. (1992). Properties of atoms in molecules: Structures and reactivities of boranes and carboranes. Can. J. Chem..

[12] Bader R.F.W. (1990). Atoms in Molecules: A Quantum Theory.

[13] Savin P.-D.A., Nesper R., Wengert S., Fässler T.F. (1997). ELF: The Electron Localization Function. Angew. Chem. Int. Ed..

[14] Morgante P., Peverati R. (2020). The devil in the details: A tutorial review on some undervalued aspects of density functional theory calculations. Int. J. Quant. Chem..

[15] Shen Y.-F., Xu C., Cheng L.-J. (2017). Deciphering chemical bonding in BnHn^2−^(n = 2–17): Flexible multicenter bonding. RSC Adv..

[16] Teixidor C.V. (2013). The uniqueness of boron as a novel challenging element for drugs in pharmacology, medicine and for smart biomaterials. Future Med. Chem..

[17] Evamarie Hey-Hawkins C.V.T. (2018). Boron-Based Compounds: Potential and Emerging Applications in Medicine.

[18] Poater J., Solà M., Viñas C., Teixidor F. (2016). Hückel’s Rule of Aromaticity Categorizes Aromatic closo Boron Hydride Clusters. Chem. A Eur. J..

[19] Duchêne L., Remhof A., Hagemann H., Battaglia C. (2020). Status and prospects of hydroborate electrolytes for all-solid-state batteries. Energy Stor. Mater..

[20] Kochnev V.K., Avdeeva V.V., Goeva L.V., Malinina E.A., Kuznetsov N.T. (2012). The undecahydrodecaborate anion B_10_H_11_—As the starting reagent in exopolyhedral substitution and complexation: Theoretical and experimental prerequisites. Russ. J. Inorg. Chem..

[21] Huang Z., Wang S., Dewhurst R.D., Ignat’Ev N.V., Finze M., Braunschweig H. (2019). Boron: Its Role in Energy—Related Processes and Applications. Angew. Chem. Int. Ed..

[22] Ali F., Hosmane N.S., Zhu Y. (2020). Boron Chemistry for Medical Applications. Molecules.

[23] Hu K., Yang Z., Zhang L., Xie L., Wang L., Xu H., Josephson L., Liang S.H., Zhang M.-R. (2020). Boron agents for neutron capture therapy. Coord. Chem. Rev..

[24] Vorobyeva M., Dymova M., Novopashina D., Kuligina E., Timoshenko V., Kolesnikov I., Taskaev S., Richter V., Venyaminova A. (2021). Tumor Cell-Specific 2′-Fluoro RNA Aptamer Conjugated with *Closo*-Dodecaborate as A Potential Agent for Boron Neutron Capture Therapy. Int. J. Mol. Sci..

[25] Lipscomb W.N. (1966). Framework Rearrangement in Boranes and Carboranes. Science.

[26] Dobrott R.D., Lipscomb W.N. (1962). Structure of Cu_2_B_10_H_10_. J. Chem. Phys..

[27] Schwalbe C.H., Lipscomb W.N. (1969). Structures of B_20_H_182_- and B_20_H_18_NO_3_—And conformations of the triethylammonium ion. J. Am. Chem. Soc..

[28] Lipscomb W.N. (1977). The Boranes and Their Relatives. Science.

[29] Hoffmann R., Lipscomb W.N. (1962). Theory of Polyhedral Molecules. I. Physical Factorizations of the Secular Equation. J. Chem. Phys..

[30] Hoffmann R., Lipscomb W.N. (1962). Theory of Polyhedral Molecules. III. Population Analyses and Reactivities for the Carboranes. J. Chem. Phys..

[31] Hoffmann R., Lipscomb W.N. (1962). Boron Hydrides: LCAO—MO and Resonance Studies. J. Chem. Phys..

[32] Wade K. (1976). Structural and Bonding Patterns in Cluster Chemistry. Adv. Inorg. Chem. Radiochem..

[33] Fox M.A., Nervi C., Crivello A., Batsanov A.S., Howard J.A.K., Wade K., Low P.J. (2009). Structural, spectroscopic, electrochemical and computational studies of C,C′-diaryl-ortho-carboranes, 1-(4-XC_6_H_4_)-2-Ph-1,2-C_2_B_10_H_10_ (X = H, F, OMe, NMe_2_, NH_2_, OH and O−). J. Solid State Electrochem..

[34] Nelyubin A.V., Selivanov N.A., Bykov A.Y., Klyukin I.N., Novikov A.S., Zhdanov A.P., Karpechenko N.Y., Grigoriev M.S., Zhizhin K.Y., Kuznetsov N.T. (2021). Primary Amine Nucleophilic Addition to Nitrilium Closo-Dodecaborate [B_12_H_11_NCCH_3_]^−^: A Simple and Effective Route to the New BNCT Drug Design. Int. J. Mol. Sci..

[35] Mingos D.M.P. (1984). Polyhedral skeletal electron pair approach. Acc. Chem. Res..

[36] Copley R.C.B., Mingos D.M.P. (1996). The novel structure of the [Au_11_(PMePh_2_)_10_]^3+^ cation: Crystal structures of [Au_11_(PMePh_2_)_10_][C_2_B_9_H_12_]_3_·4thf and [Au_11_(PMePh_2_)_10_][C_2_B_9_H_12_]_3_ (thf = tetrahydrofuran). J. Chem. Soc. Dalt. Trans..

[37] Yan Y.-K., Mingos D.M.P., Kurmoo M., Li W.-S., Scowen I.J., McPartlin M., Coomber A.T., Friend R.H. (1995). Synthesis, structure and physical properties of tetrathiafulvalenium salts of the ferracarborane complex commo-[3,3′-Fe{1-(C_4_H_3_S)-1,2-C_2_B_9_H_10_}_2_]^−^. J. Chem. Soc. Dalt. Trans..

[38] Hertler W.R., Knoth W.H., Muetterties E.L. (1965). Chemistry of Boranes. XXIV. Carbonylation of Derivatives of B_10_H_10_^2-^ and B_12_H_l2_^2-^ with Oxalyl Chloride. Inorg. Chem..

[39] Miller H.C., Miller N.E., Muetterties E.L. (1964). Chemistry of Boranes. XX. Syntheses of Polyhedral Boranes. Inorg. Chem..

[40] Chamberland B.L., Muetterties E.L. (1964). Chemistry of Boranes. XVIII. Oxidation of B_10_H_10_^-2^ and Its Derivatives. Inorg. Chem..

[41] Miller H.C., Hertler W.R., Muetterties E.L., Knoth W.H., Miller N.E. (1965). Chemistry of Boranes. XXV. Synthesis andc Chemistry of Base Derivatives of B_10_H_10_^2-^ and B_12_H_l2_^2-^. Inorg. Chem..

[42] Golub I., Filippov O., Belkova N., Epstein L., Shubina E. (2021). The Reaction of Hydrogen Halides with Tetrahydroborate Anion and Hexahydro-*closo*-hexaborate Dianion. Molecules.

[43] Černý R., Brighi M., Murgia F. (2020). The Crystal Chemistry of Inorganic Hydroborates. Chemistry.

[44] Zhao X., Yang Z., Chen H., Wang Z., Zhou X., Zhang H. (2021). Progress in three-dimensional aromatic-like closo-dodecaborate. Coord. Chem. Rev..

[45] Kamin A.A., Juhasz M.A. (2019). Exhaustive Cyanation of the Dodecaborate Dianion: Synthesis, Characterization, and X-ray Crystal Structure of [B12(CN)12]^2−^. Inorg. Chem..

[46] Lacroix M.R., Liu Y., Strauss S.H. (2019). Hydrated Metal Ion Salts of the Weakly Coordinating Fluoroanions PF_6_^−^, TiF_6_^2−^, B_12_F_12_^2−^, Ga(C_2_F_5_)_4_^−^, B(3,5-C_6_H_3_(CF_3_)_2_)_4_^−^, and Al(OC(CF_3_)_3_)_4_^−^. In Search of the Weakest HOH⋯F Hydrogen Bonds. Inorg. Chem..

[47] Klyukin I.N., Novikov A.S., Zhdanov A.P., Zhizhin K.Y., Kuznetsov N.T. (2019). QTAIM Analysis of Mono-Hydroxy Derivatives of closo-Borate Anions [B_n_H_n–1_OH]^2^ (n = 6, 10, 12). Russ. J. Inorg. Chem..

[48] Kononova E., Klemenkova Z. (2013). The electronic structure of nido-B10H14 and [6-Ph-nido-6-CB_9_H_11_] in terms of Bader’s theory (AIM). J. Mol. Struct..

[49] Mindich A.L., Bokach N.A., Dolgushin F.M., Haukka M., Lisitsyn L.A., Zhdanov A.P., Zhizhin K.Y., Miltsov S.A., Kuznetsov N.T., Kukushkin V.Y. (2012). 1,3-Dipolar Cycloaddition of Nitrones to a Nitrile Functionality in closo-Decaborate Clusters: A Novel Reactivity Mode for the Borylated C≡N Group. Organometallics.

[50] Burianova V.K., Mikherdov A.S., Bolotin D.S., Novikov A.S., Mokolokolo P.P., Roodt A., Boyarskiy V.P., Suslonov V.V., Zhdanov A.P., Zhizhin K.Y. (2018). Electrophilicity of aliphatic nitrilium closo-decaborate clusters: Hyperconjugation provides an unexpected inverse reactivity order. J. Organomet. Chem..

[51] Bolotin D.S., Burianova V.K., Novikov A.S., Demakova M.Y., Pretorius C., Mokolokolo P.P., Roodt A., Bokach N.A., Suslonov V.V., Zhdanov A.P. (2016). Nucleophilicity of Oximes Based upon Addition to a Nitrilium closo-Decaborate Cluster. Organometallics.

[52] Stogniy M.Y., Erokhina S.A., Sivaev I.B., Bregadze V.I. (2019). Nitrilium derivatives of polyhedral boron compounds (boranes, carboranes, metallocarboranes): Synthesis and reactivity. Phosph. Sulf. Silicon Relat. Elem..

[53] Golub I.E., Filippov O.A., Belkova N.V., Epstein L.M., Shubina E.S. (2021). The Mechanism of Halogenation of Decahydro-closo-Decaborate Dianion by Hydrogen Chloride. Russ. J. Inorg. Chem..

[54] Shore S.G., Hamilton E.J.M., Bridges A.N., Bausch J., Krause-Bauer J.A., Dou D., Liu J., Liu S., Bin D., Hall H. (2003). The Solid State Structure of [B_10_H_11_]^−^ and Its Dynamic NMR Spectra in Solution. Inorg. Chem..

[55] Kochnev V.K., Avdeeva V.V., Malinina E.A., Kuznetsov N.T. (2014). Theoretical study of H2 elimination from [B_n_H_n+1_] monoanions (n = 6–9, 11). Russ. J. Inorg. Chem..

[56] Sun Y., Zhang J., Zhang Y., Liu J., Van Der Veen S., Duttwyler S. (2018). The closo-Dodecaborate Dianion Fused with Oxazoles Provides 3D Diboraheterocycles with Selective Antimicrobial Activity. Chem. A Eur. J..

[57] Cao K., Zhang C.-Y., Xu T.-T., Wu J., Wen X.-Y., Jiang W.-J., Chen M., Yang J. (2020). Synthesis of Polyhedral Borane Cluster Fused Heterocycles via Transition Metal Catalyzed B-H Activation. Molecules.

[58] Zhu T.-C., Xing Y.-Y., Sun Y., Duttwyler S., Hong X. (2020). Directed B–H functionalization of the closo-dodecaborate cluster via concerted iodination—deprotonation: Reaction mechanism and origins of regioselectivity. Org. Chem. Front..

[59] Voinova V.V., Selivanov N.A., Plyushchenko I.V., Vokuev M.F., Bykov A.Y., Klyukin I.N., Novikov A.S., Zhdanov A.P., Grigoriev M.S., Rodin I.A. (2021). Anion—Novel Members of Diboroheterocycle Class. Molecules.

[60] Klyukin I., Zhdanov A., Matveev E., Razgonyaeva G., Grigoriev M., Zhizhin K., Kuznetsov N. (2014). Synthesis and reactivity of closo -decaborate anion derivatives with multiple carbon-oxygen bonds. Inorg. Chem. Commun..

[61] Laskova J., Ananiev I., Kosenko I., Serdyukov A., Stogniy M.Y., Sivaev I.B., Grin M.A., Semioshkin A., Bregadze V.I. (2022). Nucleophilic addition reactions to nitrilium derivatives [B_12_H_11_NCCH_3_]^−^ and [B_12_H_11_NCCH_2_CH_3_]. Synthesis and structures of closo-dodecaborate-based iminols, amides and amidines. Dalt. Trans..

[62] Avdeeva V.V., Polyakova I.N., Goeva L.V., Malinina E.A., Kuznetsov N.T. (2014). [2,6(9)-B_10_H_8_(O)_2_CCH_3_]^−^ and [2,7(8)- B_10_H_8_ (OC(O)CH_3_)_2_]^2−^ derivatives in synthesis of position isomers of the [B_10_H_8_(OC(O)CH_3_)(OH)]^2−^ anion with the 2,6(9)- and 2,7(8)-arrangement of functional groups. Russ. J. Inorg. Chem..

[63] Klyukin I., Kubasov A., Limarev I., Zhdanov A., Matveev E., Polyakova I., Zhizhin K., Kuznetsov N. (2015). The new approach to formation of exo boron-oxygen bonds from the decahydro-closo-decaborate(2-) anion. Polyhedron.

[64] Oller J., Pérez P., Ayers P.W., Vöhringer-Martinez E. (2018). Global and local reactivity descriptors based on quadratic and linear energy models forα,β-unsaturated organic compounds. Int. J. Quant. Chem..

[65] Gómez T., Fuentealba P., Robles-Navarro A., Cárdenas C. (2021). Links among the Fukui potential, the alchemical hardness and the local hardness of an atom in a molecule. J. Comput. Chem..

[66] Khanum G., Ali A., Shabbir S., Fatima A., Alsaiari N., Fatima Y., Ahmad M., Siddiqui N., Javed S., Gupta M. (2022). Vibrational Spectroscopy, Quantum Computational and Molecular Docking Studies on 2-[(1H-Benzimidazol-1-yl)-methyl]benzoic Acid. Crystals.

[67] Klyukin I.N., Vlasova Y.S., Novikov A.S., Zhdanov A.P., Hagemann H.R., Zhizhin K.Y., Kuznetsov N.T. (2022). B-F bonding and reactivity analysis of mono- and perfluoro-substituted derivatives of closo-borate anions (6, 10, 12): A computational study. Polyhedron.

[68] Klyukin I., Vlasova Y., Novikov A., Zhdanov A., Zhizhin K., Kuznetsov N. (2021). Theoretical Study of *closo*-Borate Anions [B_n_H_n_]^2−^ (*n* = 5–12): Bonding, Atomic Charges, and Reactivity Analysis. Symmetry.

[69] Hehre W.J., Ditchfield R., Pople J.A. (1972). Self—Consistent Molecular Orbital Methods. XII. Further Extensions of Gaussian—Type Basis Sets for Use in Molecular Orbital Studies of Organic Molecules. J. Chem. Phys..

[70] Grimme S., Antony J., Ehrlich S., Krieg H. (2010). A consistent and accurate ab initio parametrization of density functional dispersion correction (DFT-D) for the 94 elements H-Pu. J. Chem. Phys..

[71] Neese F. (2012). The ORCA program system. Wiley Interdiscip. Rev. Comput. Mol. Sci..

[72] Gonzalez C., Schlegel H.B. (1991). Improved algorithms for reaction path following: Higher-order implicit algorithms. J. Chem. Phys..

[73] Gonzalez C., Schlegel H.B. (1989). An improved algorithm for reaction path following. J. Chem. Phys..

[74] Gonzalez C., Schlegel H.B. (1990). Reaction path following in mass-weighted internal coordinates. J. Phys. Chem..

[75] Marenich A.V., Cramer C.J., Truhlar D.G. (2009). Universal Solvation Model Based on Solute Electron Density and on a Continuum Model of the Solvent Defined by the Bulk Dielectric Constant and Atomic Surface Tensions. J. Phys. Chem. B.

[76] Glendening E.D., Landis C.R., Weinhold F. (2019). NBO 7.0: New vistas in localized and delocalized chemical bonding theory. J. Comput. Chem..

[77] Lu T., Chen F. (2011). Multiwfn: A multifunctional wavefunction analyzer. J. Comput. Chem..

[78] Adrienko G.A. (2017). Chemcraft, Version 1.8 (Build 530). http://www.chemcraftprog.com.

