# Supplementary material for: Protonation of Borylated Carboxonium Derivative [2,6-B10H8O2CCH3]−: Theoretical and Experimental Investigation
Source: Int J Mol Sci. 2022 Apr 10;23(8):4190. doi: 10.3390/ijms23084190 (PMC9025682; doi:10.3390/ijms23084190)
Supplement: Supplementary file 1 [file ijms-23-04190-s001.zip › ijms-1646891-supplementary.pdf]

Supplementary materials

Protonation of borylated carboxonium derivative [2,6-$B_{10}H_8O_2CCH_3$]$^-$: theoretical and experimental investigation

Ilya N. Klyukin[1*], Anastasia V. Kolbunova[1,2], Alexander S. Novikov[3], Aleksey V. Nelyubin[1], Nikita A. Selivanov[1], Alexander Yu. Bykov[1], Alexandra A. Klyukina[4], Andrey P. Zhdanov[1], Konstantin Yu. Zhizhin[1,5], Nikolay T. Kuznetsov[1]

1) Kurnakov Institute of General and Inorganic Chemistry,
Russian Academy of Sciences,
Leninskii pr. 31, 117907, Moscow, Russian Federation;

2) Higher School of Economics, Faculty of Chemistry,
Myasnitskaya yl. 101000, Moscow, Russian Federation;

3) Saint Petersburg State University, Institute of Chemistry,
Universitetskaya Nab. 7-9, 199034, Saint Petersburg, Russian Federation

4) Winogradsky Institute of Microbiology, Research Centre of Biotechnology,
Russian Academy of Sciences,
60 let Oktjabrja pr-t, 7, bld. 2, 117312 Moscow, Russia.

5) MIREA Russian Technological University, Lomonosov Institute of Fine Chemical Technologies, 119571, Moscow, Russia

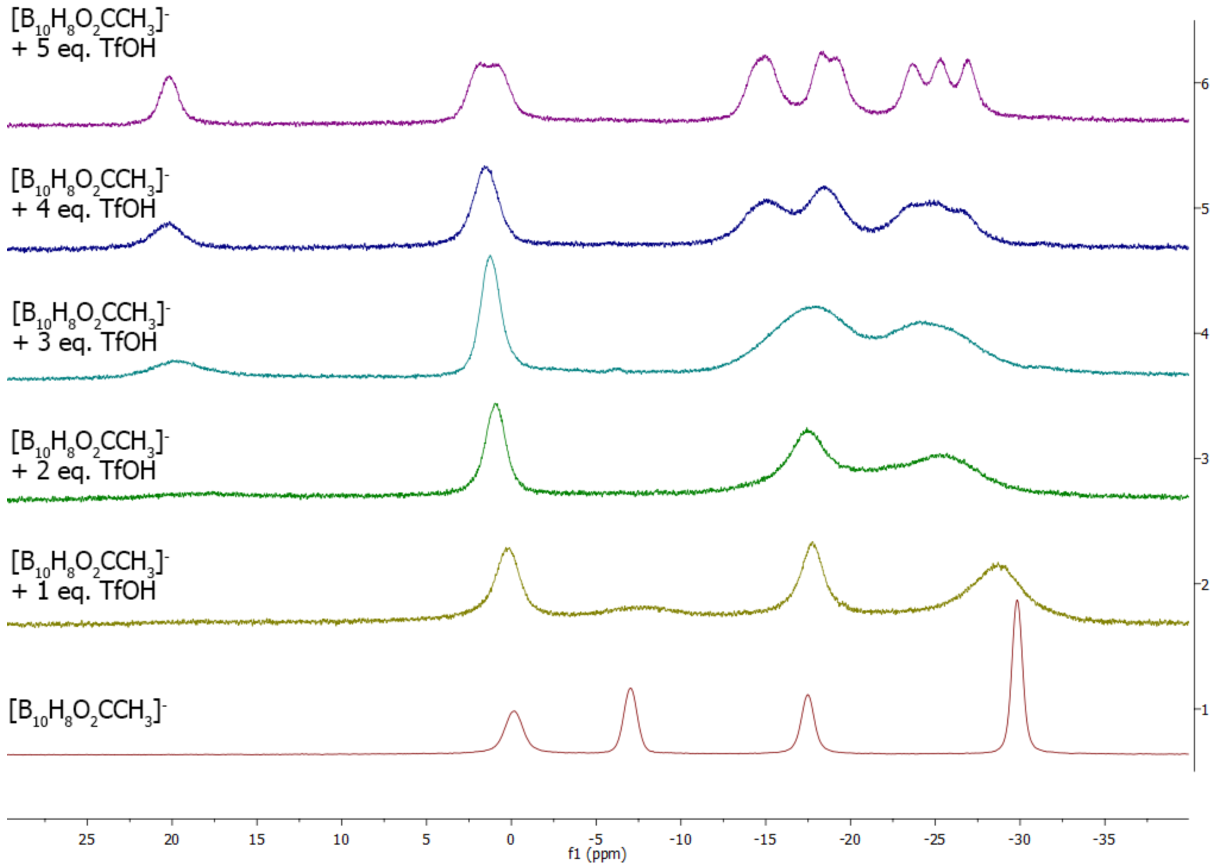

Figure S1. ^{11}B-^{1}H spectra of [2,6-$B_{10}H_8O_2CCH_3$]$^-$ with presence of different amount of CF_3SO_3H acid.

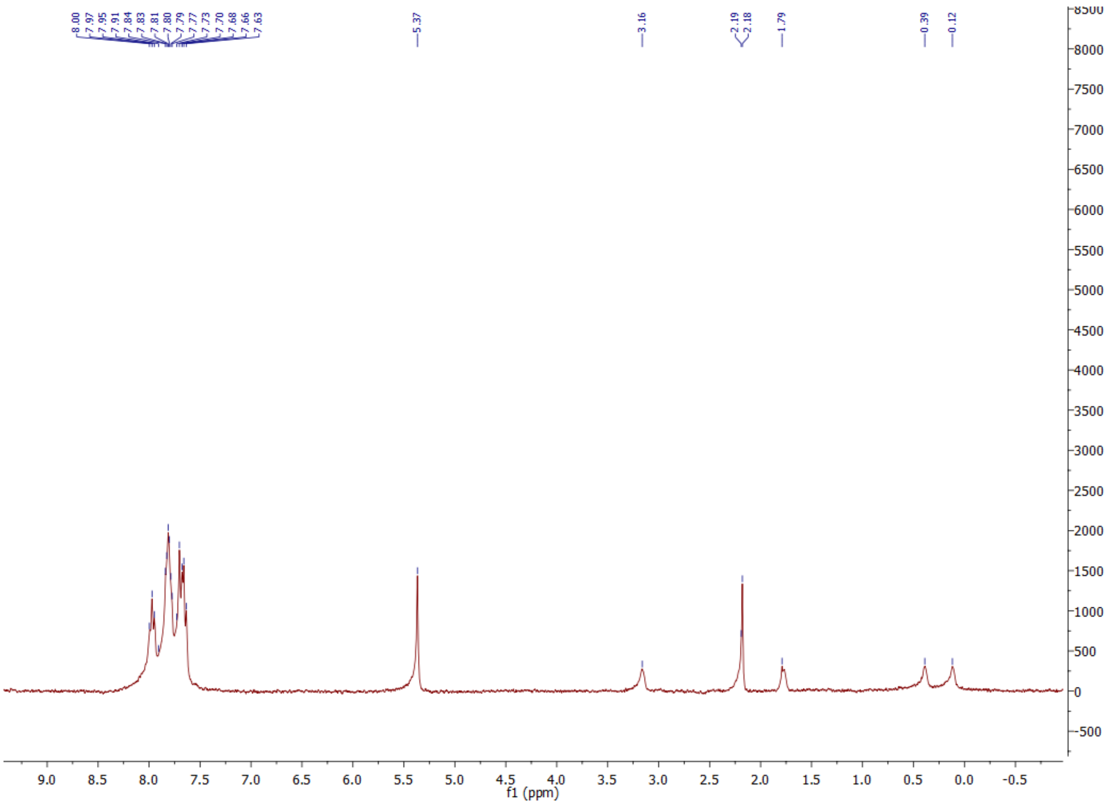

Figure S2. 1H-11B spectra of [2,6-$B_{10}H_8O_2CCH_3$]$^-$.

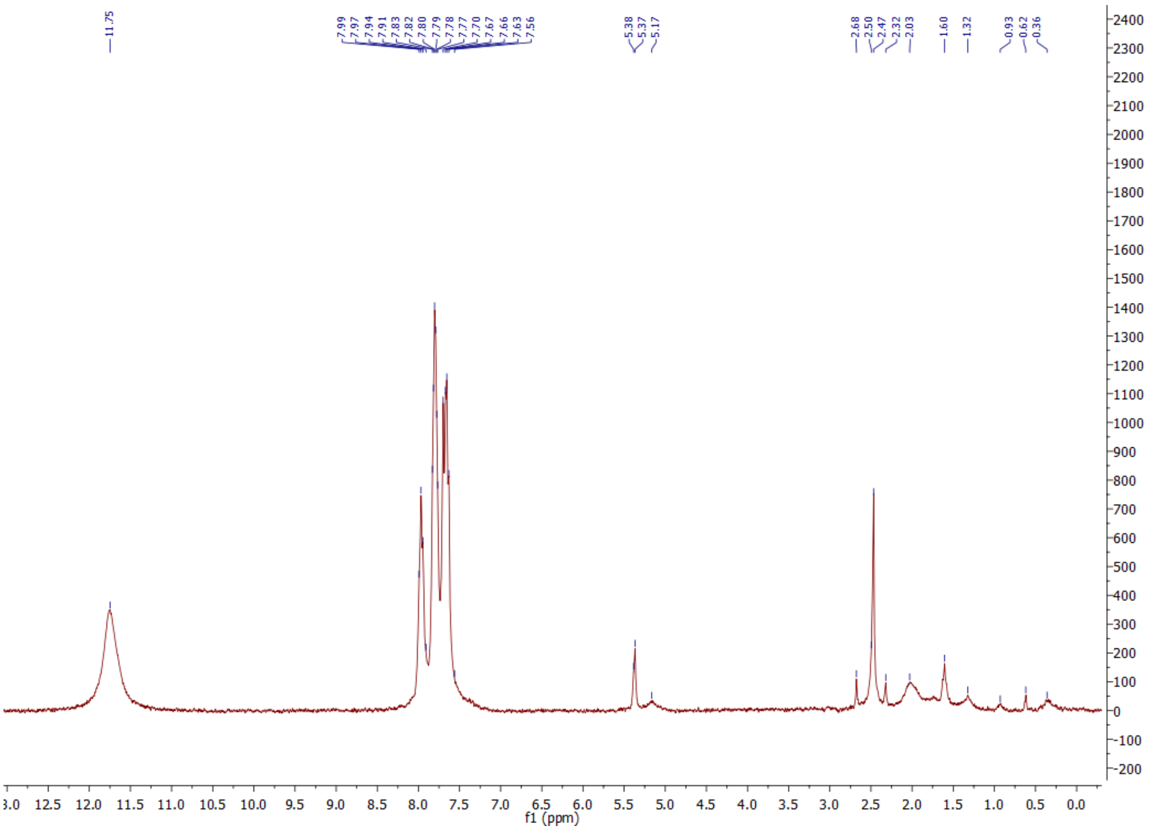

Figure S3. ^{1}H-^{11}B spectra of $[2.6\text{-}B_{10}H_8O_2CCH_3{*}H^{fac}]^-$.

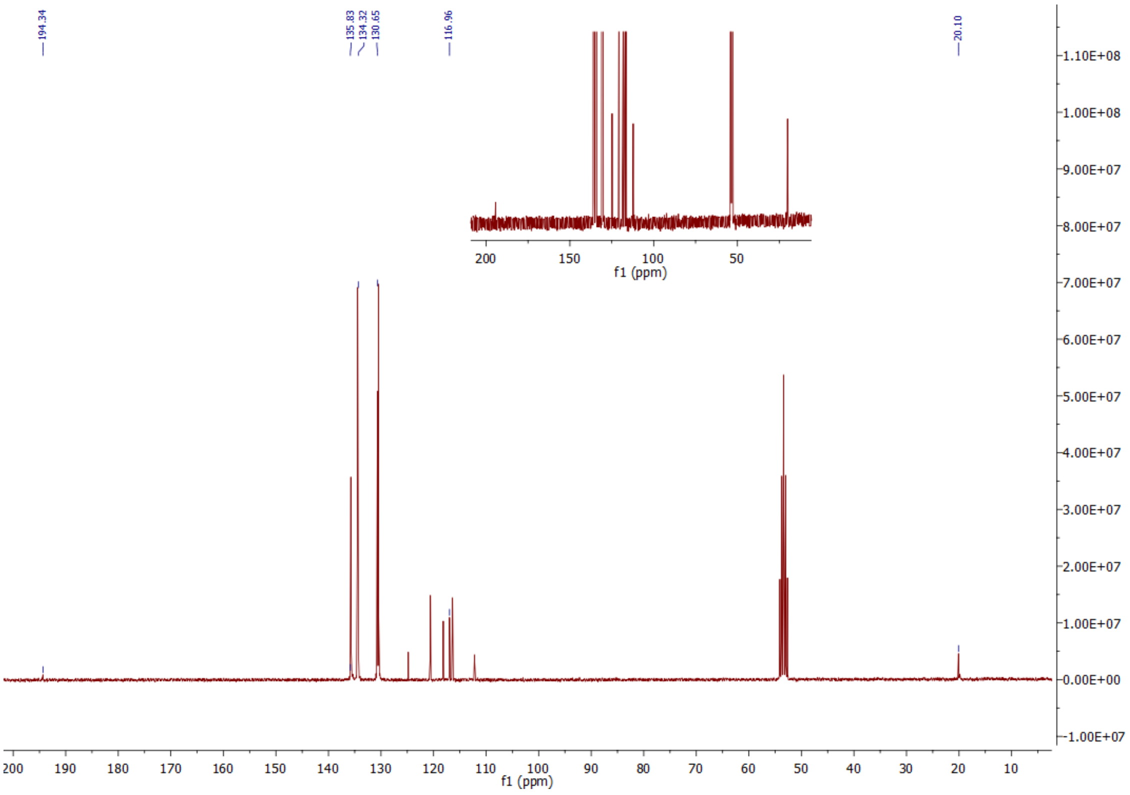

Figure S4. ^{13}C spectra of $[2.6\text{-}B_{10}H_8O_2CCH_3{*}H^{fac}]^-$.

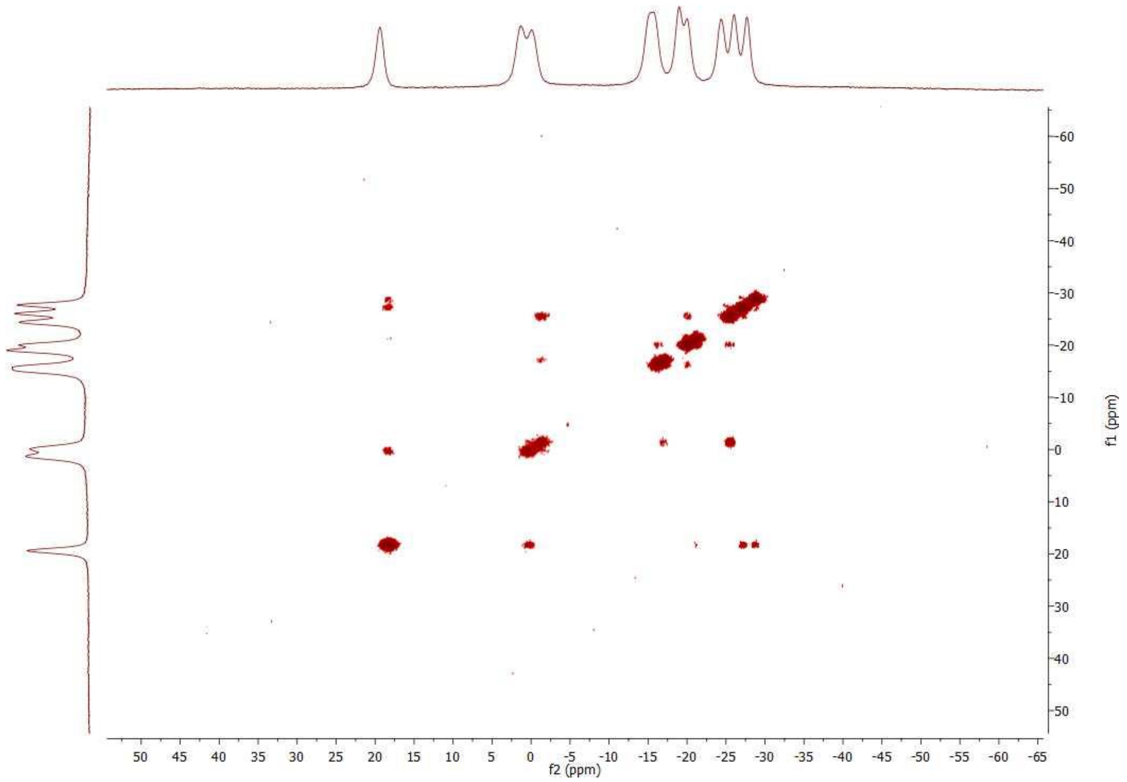

Figure S5. ^{11}B-^{11}B spectra of [2.6-$B_{10}H_8O_2CCH_3$*H^{fac}]$^-$.

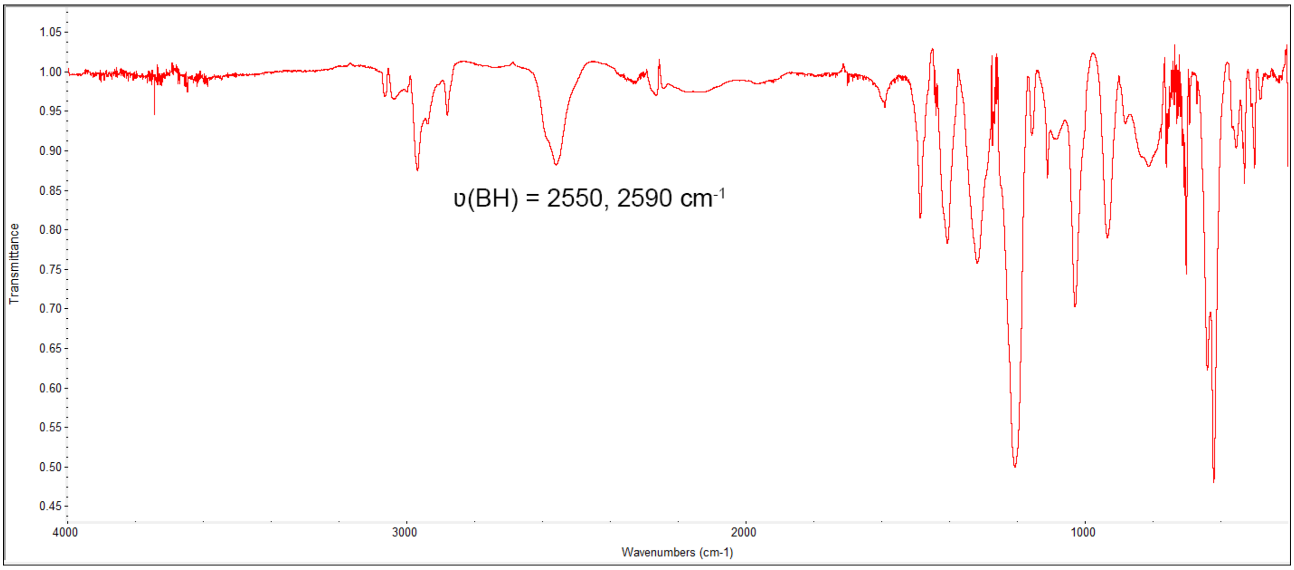

Figure S6. IR-spectra of [2.6-$B_{10}H_8O_2CCH_3$*H^{fac}]$^-$.

Table S1. B–X bond lengths**,** Wiberg indices, and main topological parameters of electron density for B–F interactions. *ρ(r)* – electron density at the bcp, $\nabla^2\rho(r)$ – Laplacian of electron density at the bcp, H_b – total energy at the bcp, δ(B–X) – delocalization index.

	B–X length (Å)	$\rho(r)$ (e Å^{-3})	$\nabla^2\rho(r)$ (e Å^{-5})	H_b (h e^{-1})	δ(B–H)	Wiberg index
			$[B_{10}H_8O_2CCH_3]^-$			
B1-H	1.196	0.166	-0.136	-0.166	0.721	0.95
B3-H	1.199	0.169	-0.177	-0.172	0.704	0.95
B4-H	1.200	0.168	-0.168	-0.171	0.701	0.95
B5-H	1.203	0.165	-0.147	-0.166	0.702	0.94
B7-H	1.203	0.165	-0.147	-0.167	0.701	0.94
B8-H	1.200	0.168	-0.167	-0.171	0.702	0.95
B9-H	1.199	0.169	-0.178	-0.172	0.704	0.95
B10-H	1.196	0.166	-0.136	-0.166	0.722	0.95
B2-O	1.539	0.121	0.579	-0.067	0.495	0.61
B6-O	1.539	0.121	0.578	-0.067	0.494	0.61
			$[B_{10}H_8O_2CCH_3{*}H^{fac}]^0$			
B1-H^{fac}	1.286	0.129	0.101	-0.116	0.447	0.35
B3-H^{fac}	1.420	0.109	-0.081	-0.083	0.331	0.25
B1-H	1.186	0.177	-0.235	-0.184	0.702	0.90
B3-H	1.190	0.181	-0.286	-0.193	0.675	0.94
B4-H	1.188	0.181	-0.267	-0.191	0.698	0.95
B5-H	1.193	0.175	-0.215	-0.181	0.708	0.91
B7-H	1.193	0.174	-0.212	-0.180	0.711	0.93
B8-H	1.192	0.176	-0.223	-0.183	0.713	0.94
B9-H	1.190	0.177	-0.235	-0.184	0.718	0.93
B10-H	1.186	0.176	-0.215	-0.181	0.730	0.93
B2-O	1.502	0.138	0.647	-0.080	0.512	0.63
B6-O	1.511	0.134	0.626	-0.076	0.501	0.64
			$[B_{10}H_{11}]^-$			
B1-H^{fac}	1.301	0.124	0.133	-0.109	0.430	0.39
B1-H	1.192	0.172	-0.187	-0.176	0.690	0.94
B2-H	1.197	0.174	-0.223	-0.181	0.678	0.95
B3-H	1.197	0.174	-0.228	-0.182	0.681	0.95
B4-H	1.198	0.170	-0.184	-0.174	0.699	0.94
B5-H	1.198	0.170	-0.186	-0.174	0.702	0.94
B6-H	1.199	0.169	-0.184	-0.173	0.707	0.94
B7-H	1.201	0.169	-0.178	-0.173	0.708	0.95

B8-H	1.199	0.169	-0.180	-0.173	0.706	0.94
B9-H	1.199	0.169	-0.184	-0.173	0.704	0.95
B10-H	1.195	0.169	-0.162	-0.171	0.722	0.95

Table S2. NBO, QTAIM and Hirshfeld atomic charges of derivative $[B_{10}H_8O_2CCH_3]^-$ in a gas phase.

	Hirshfeld	NBO	QTAIM
1O	-0.09	-0.57	-1.31
2O	-0.09	-0.58	-1.31
3C	0.29	0.92	1.63
4C	-0.07	-0.76	0.08
5H	0.08	0.29	0.07
6H	0.07	0.28	0.06
7H	0.07	0.28	0.06
8B	-0.01	-0.09	0.47
9H	-0.03	0.07	-0.58
10B	0.07	0.34	0.31
11B	-0.01	-0.17	0.66
12H	-0.04	0.09	-0.59
13B	0.01	-0.09	0.67
14H	-0.03	0.08	-0.60
15B	-0.05	-0.25	0.62
16H	-0.04	0.09	-0.59
17B	0.07	0.34	0.30
18B	-0.05	-0.25	0.62
19H	-0.04	0.09	-0.59
20B	0.01	-0.09	0.67
21H	-0.03	0.08	-0.60
22B	-0.01	-0.17	0.66
23H	-0.04	0.09	-0.59
24B	-0.01	-0.09	0.47
25H	-0.03	0.07	-0.58

Table S3. Fukui function values based on NBO, QTAIM, and Hirshfeld atomic charges of derivative $[B_{10}H_8O_2CCH_3]^-$ in a gas phase.

	Hirshfeld	NBO	QTAIM
1O	0.02	0.01	0.00
2O	0.02	0.01	0.00

3C	0.02	0.02	-0.03
4C	0.01	-0.01	0.01
5H	0.02	0.02	0.03
6H	0.02	0.02	0.03
7H	0.02	0.02	0.03
8B	0.10	0.17	-0.10
9H	0.05	0.04	0.06
10B	0.05	0.09	0.20
11B	0.03	-0.01	0.05
12H	0.04	0.04	0.05
13B	0.06	0.09	0.07
14H	0.04	0.03	0.05
15B	0.03	-0.02	0.05
16H	0.04	0.04	0.05
17B	0.05	0.08	0.20
18B	0.03	-0.02	0.05
19H	0.04	0.04	0.05
20B	0.06	0.09	0.08
21H	0.04	0.03	0.05
22B	0.03	-0.01	0.05
23H	0.04	0.04	0.05
24B	0.10	0.16	-0.12
25H	0.05	0.03	0.06

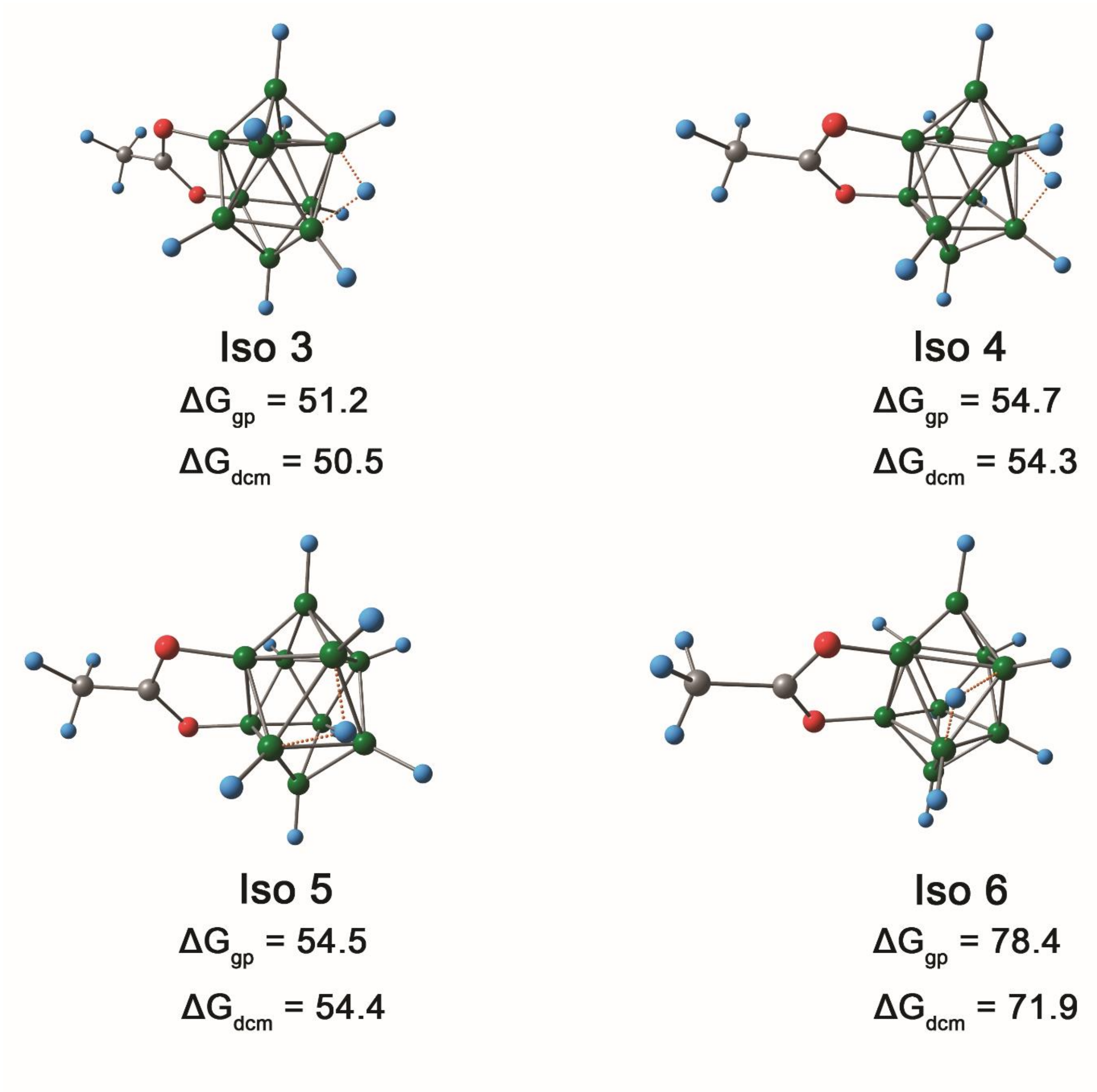

Figure S7. Optimized structures for equatorial isomer of $[B_{10}H_8O_2CCH_3{*}H^{fac}]^0$. Atomic designations were given in Figure 1 of the main text.

Table S4. Relative Gibbs energy of $[B_{10}H_8O_2CCH_3{*}H^{fac}]^0$ isomers.

	Gas phase	CH_2Cl_2
Iso1	0.0	0.0
TS1	28.9	30.8
Iso2	21.7	22.3
TS2	80.1	80.3
Iso3	51.2	50.5
Iso4	54.7	54.3
Iso5	54.5	54.4

Iso6	78.4	71.9

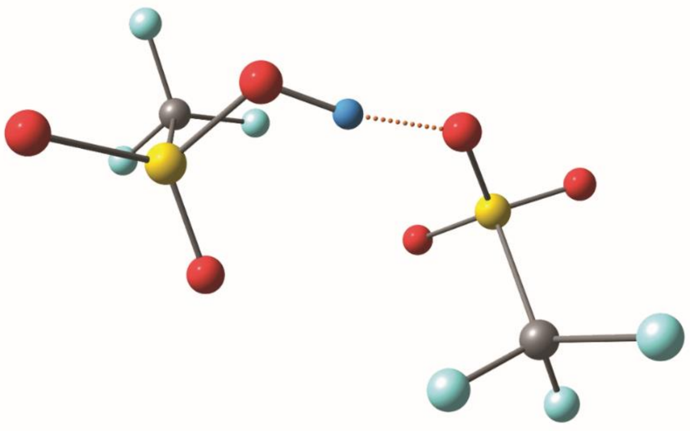

Figure S8. Optimized structures for supramolecular associates of $CF_3SO_3^-$ with CF_3SO_3H. Atomic designations were given in Figure 1 of the main text.

Table S5. Predicted ^{11}B NMR chemical shifts (in ppm) for $[B_{10}H_8O_2CCH_3]^-$ and $[B_{10}H_8O_2CCH_3{*}H^{fac}]^0$. σ_{ref} - absolute isotropic shielding for ^{11}B nuclei of BF_3Et_2O. δ_{theor} – relative shifts of ^{11}B nuclei (in ppm). ($\delta_{theor} = \sigma_{ref} - \sigma_{abs}$, σ_{abs} – absolute, isotropic shielding for the ^{11}B nuclei). $\Delta = \delta_{exp} - \delta_{theor}$ (for values δ_{exp} see Experimental Section).

$[B_{10}H_8O_2CCH_3]^-$

B97						
	IGLOIII		EPR-III		6-31++G(d,p)	
	σ_{ref}	99.66	σ_{ref}	100.336	σ_{ref}	110.85
	δ_{theor}	Δ	δ_{theor}	Δ	δ_{theor}	Δ
B1	-11.8	4.7	-12.6	5.5	-11.5	4.4
B2	-1.3	1.3	-2.3	2.3	-2.7	2.7
B3	-22.3	4.7	-23.8	6.2	-22.3	4.7
B4	-34.2	4.2	-34.7	4.7	-32.6	2.6
B5	-33.5	3.5	-32.9	2.9	-33.1	3.1
B6	-1.4	1.4	-1.5	1.5	-2.0	2.0
B7	-33.7	3.7	-33.8	3.8	-31.0	1.0
B8	-34.2	4.2	-34.2	4.2	-31.4	1.4
B9	-21.6	4.0	-18.8	1.2	-19.5	1.9
B10	-10.3	3.2	-11.2	4.1	-16.3	9.2
	RMSD	3.69	RMSD	3.97	RMSD	4.01
B3LYP						
	IGLOIII		EPR-III		6-31++G(d,p)	
	σ_{ref}	97.616	σ_{ref}	97.909	σ_{ref}	109.532

	δ_{theor}	Δ	δ_{theor}	Δ	δ_{theor}	Δ
B1	-11.6	4.5	-12.7	5.6	-11.6	4.5
B2	-2.6	2.6	-3.9	3.9	-4.0	4.0
B3	-22.7	5.1	-24.8	7.2	-23.0	5.4
B4	-34.5	4.5	-35.3	5.3	-32.8	2.8
B5	-34.3	4.3	-33.6	3.6	-33.7	3.7
B6	-2.7	2.7	-3.0	3.0	-3.2	3.2
B7	-34.7	4.7	-35.2	5.2	-31.9	1.9
B8	-34.4	4.4	-34.5	4.5	-31.7	1.7
B9	-21.9	4.3	-18.8	1.2	-20.7	3.1
B10	-10.1	3.0	-11.2	4.1	-16.0	8.9
	RMSD	4.10	RMSD	4.64	RMSD	4.38
wB97X-D3						
	IGLOIII		EPR-III		6-31++G(d,p)	
	σ_{ref}	120.286	σ_{ref}	119.279	σ_{ref}	129.015
	δ_{theor}	Δ	δ_{theor}	Δ	δ_{theor}	Δ
B1	-93.4	86.3	-92.7	85.6	-67.0	59.9
B2	-52.8	52.8	-58.5	58.5	-43.2	43.2
B3	-101.9	84.3	-97.3	79.7	-104.8	87.2
B4	-70.8	40.8	-75.9	45.9	-81.2	51.2
B5	-39.1	9.1	-46.5	16.5	-67.9	37.9
B6	6.4	-6.4	5.3	-5.3	-22.7	22.7
B7	-68.2	38.2	-69.1	39.1	-56.8	26.8
B8	-62.8	32.8	-61.5	31.5	-34.5	4.5
B9	-3.7	-13.9	-11.4	-6.2	1.0	-18.6
B10	16.5	-23.6	10.9	-18.0	-63.9	56.8
	RMSD	47.34	RMSD	47.31	RMSD	46.84

$[B_{10}H_8O_2CCH_3{*}H^{fac}]^0$

B97						
	IGLOIII		EPR-III		6-31++G(d,p)	
	σ_{ref}	99.66	σ_{ref}	100.336	σ_{ref}	110.85
	δ_{theor}	Δ	δ_{theor}	Δ	δ_{theor}	Δ
B1	13,4	5,8	13,0	6,2	10,1	9,1
B2	0,3	1,1	0,2	1,2	-0,9	2,3
B3	-23,7	3,6	-24,2	4,1	-23,5	3,4
B4	-32,4	4,6	-32,7	4,9	-30,9	3,1
B5	-28,6	4,2	-28,7	4,3	-27,9	3,5
B6	-2,5	2,3	-2,7	2,5	-3,3	3,1
B7	-22,9	3,8	-23,0	3,9	-22,7	3,6
B8	-16,5	1,3	-16,8	1,6	-16,7	1,5
B9	-20,3	4,6	-20,1	4,4	-20,0	4,3
B10	-27,3	1,2	-27,2	1,1	-26,6	0,5
	RMSD	3,61	RMSD	3,78	RMSD	4,06
B3LYP						

	IGLOIII		EPR-III		6-31++G(d,p)	
	σ_{ref}	97.616	σ_{ref}	97.909	σ_{ref}	109.532
	δ_{theor}	Δ	δ_{theor}	Δ	δ_{theor}	Δ
B1	14,5	4,7	13,9	5,3	10,8	8,4
B2	0,8	0,6	-0,3	1,7	-1,2	2,6
B3	-24,7	4,6	-25,0	4,9	-24,2	4,1
B4	-32,6	4,8	-33,1	5,3	-31,2	3,4
B5	-28,9	4,5	-29,5	5,1	-28,4	4,0
B6	-2,1	1,9	-3,0	2,8	-3,4	3,2
B7	-22,9	3,8	-23,5	4,4	-22,9	3,8
B8	-15,8	0,6	-16,9	1,7	-16,8	1,6
B9	-20,6	4,9	-21,1	5,4	-20,8	5,1
B10	-28,4	2,3	-28,5	2,4	-27,7	1,6
	RMSD	3,67	RMSD	4,17	RMSD	4,22
wB97X-D3						
	IGLOIII		EPR-III		6-31++G(d,p)	
	σ_{ref}	120.286	σ_{ref}	119.279	σ_{ref}	129.015
	δ_{theor}	Δ	δ_{theor}	Δ	δ_{theor}	Δ
B1	-82,1	101,3	-78,9	98,1	-62,0	81,2
B2	-51,1	52,5	-59,6	61,0	-50,1	51,5
B3	-99,5	79,4	-94,0	73,9	-102,4	82,3
B4	-74,3	46,5	-78,8	51,0	-71,8	44,0
B5	-32,2	7,8	-39,3	14,9	-60,6	36,2
B6	13,5	-13,7	19,4	-19,6	10,5	-10,7
B7	-55,2	36,1	-60,7	41,6	-61,2	42,1
B8	-35,4	20,2	-38,3	23,1	-40,1	24,9
B9	-0,1	-15,6	3,7	-19,4	30,5	-46,2
B10	2,9	-29,0	6,9	-33,0	2,6	-28,7
	RMSD	49,54	RMSD	50,75	RMSD	49,71

Table S6. Predicted ^{1}H NMR chemical shifts (in ppm) of $[B_{10}H_8O_2CCH_3]^-$ and $[B_{10}H_8O_2CCH_3{*}H^{fac}]^0$. σ_{ref} - absolute isotropic shielding for ^{1}H nuclei of $Si(CH_3)_4$. δ_{theor} – relative shifts of ^{1}H nuclei (in ppm). ($\delta_{theor} = \sigma_{ref} - \sigma_{abs}$, σ_{abs} – absolute, isotropic shielding for the ^{1}H nuclei). $\Delta = \delta_{exp} - \delta_{theor}$ (for values δ_{exp} see Experimental Section).

$[B_{10}H_8O_2CCH_3]^-$		
	σ_{ref}	31.645
	δ_{theor}	Δ
Hsub	2.22	-0.04
Hsub	2.03	0.15
Hsub	2.06	0.12
H-B1	3.22	-0.05
H-B3	1.85	-0.06
H-B4	0.14	-0.01
H-B5	0.58	-0.18

H-B7	0.52	-0.12
H-B8	0.10	0.03
H-B9	1.84	-0.07
H-B10	3.32	-0.15
	RMSD	0.10
**$[B_{10}H_8O_2CCH_3{*}H^{fac}]^0$**		
	σ_{ref}	31.645
	δ_{theor}	Δ
Hsub	2.56	-0.05
Hsub	2.33	0.17
Hsub	2.41	0.09
H-B10	5.05	0.12
H-B9	2.01	0.02
H-B8	0.31	0.31
H-B7	1.32	-0.39
H-B5	1.71	-0.39
H-B4	1.97	-0.37
H-B3	2.82	-0.14
Hfac	0.00	0.36
H-B10	3.57	-
	RMSD	0.25

Table S7. Cartesian atomic coordinates of the calculated optimized equilibrium model structures. All coordinates are given in Angstrom units.

Optimized equilibrium model structures in gas phase

Compound	Atom	x	y	z
$[B_{10}H_8O_2CCH_3]^-$				
	O	7.323323	6.693397	11.200566
	O	6.598392	5.106300	9.817883
	C	7.348760	5.493732	10.772084
	C	8.259573	4.508696	11.425461
	H	7.712111	4.016671	12.235250
	H	9.121702	5.023039	11.850208
	H	8.569701	3.751522	10.705035

	B	5.591706	6.839463	7.720140
	H	5.803420	6.337506	6.655799
	B	5.772992	6.289956	9.282864
	B	4.167753	6.872505	8.689193
	H	3.170978	6.319861	8.317004
	B	5.031763	8.398381	8.141709
	H	4.775885	9.174678	7.262820
	B	6.618203	7.775719	8.722910
	H	7.777071	7.947841	8.449933
	B	6.232026	7.490820	10.464614
	B	4.542717	6.879980	10.467007
	H	4.025107	6.048157	11.165767
	B	4.037969	8.414123	9.671747
	H	2.983728	8.986760	9.686216
	B	5.760686	9.054905	9.689166
	H	6.221037	10.161733	9.707012
	B	4.992133	8.412361	11.089445
	H	4.804336	8.888334	12.170177
$[B_{10}H_8O_2CCH_3*H^{fac}]^0$ Iso 1				
	O	7.284772	6.732598	11.191875
	O	6.586260	5.131385	9.819067
	C	7.328701	5.516856	10.784968
	C	8.225695	4.546998	11.460542
	H	7.648080	4.027423	12.231758
	H	9.054371	5.070063	11.936175
	H	8.583104	3.808489	10.742982
	B	5.579422	6.852642	7.688898
	H	5.782914	6.406740	6.608848
	B	5.784455	6.284251	9.261979

	B	4.176260	6.811623	8.656978
	H	3.179824	6.278657	8.283957
	B	4.985632	8.398051	8.154607
	H	4.728563	9.170187	7.284128
	B	6.615330	7.744265	8.691029
	H	7.747883	7.983327	8.402589
	B	6.252766	7.502641	10.418605
	B	4.530412	6.885824	10.419424
	H	4.059218	6.113114	11.196303
	B	3.931961	8.327613	9.626274
	H	2.911816	8.936837	9.651260
	B	5.844747	9.067237	9.669554
	H	6.320571	10.155043	9.589101
	B	4.994329	8.385546	11.119999
	H	4.849173	8.681641	12.259320
	H	4.768137	9.486214	10.495545
$[B_{10}H_8O_2CCH_3{*}H^{fac}]^0$ TS1				
	O	7.218384	6.666966	11.259451
	O	6.620197	5.105223	9.799643
	C	7.327277	5.470927	10.794145
	C	8.283495	4.531996	11.429450
	H	8.060720	4.461664	12.496755
	H	9.294655	4.934732	11.325244
	H	8.218164	3.552676	10.959318
	B	5.671044	6.840215	7.710491
	H	5.989640	6.383766	6.662793
	B	5.789048	6.254486	9.261403
	B	4.191603	6.823726	8.614164
	H	3.217957	6.250855	8.241046

	B	4.994448	8.347980	8.077569
	H	4.800503	9.131901	7.202486
	B	6.613203	7.786937	8.801692
	H	7.762677	7.994128	8.550548
	B	6.187421	7.411348	10.488681
	B	4.537254	6.846935	10.400844
	H	3.988880	6.089773	11.140813
	B	3.973603	8.359453	9.558079
	H	2.902589	8.877404	9.572527
	B	5.626334	9.119716	9.615647
	H	6.106447	10.206385	9.513607
	B	4.832519	8.409592	11.063825
	H	4.425073	8.753147	12.124258
	H	5.911568	9.099249	10.994838
$[B_{10}H_8O_2CCH_3{*}H^{fac}]^0$ Iso 2				
	O	7.301352	6.666872	11.219967
	O	6.572013	5.117904	9.814224
	C	7.344135	5.464799	10.763645
	C	8.276641	4.485192	11.371679
	H	7.760220	3.992366	12.201668
	H	9.154185	4.997851	11.764723
	H	8.554767	3.730173	10.637108
	B	5.631230	6.820632	7.744730
	H	5.915979	6.284582	6.725048
	B	5.753274	6.279295	9.308067
	B	4.182671	6.846745	8.703456
	H	3.182628	6.289454	8.379999
	B	5.016634	8.359955	8.069975
	H	4.790403	9.106468	7.169165

	B	6.607828	7.820839	8.757939
	H	7.758174	7.982513	8.477939
	B	6.246372	7.470222	10.530573
	B	4.556769	6.896773	10.468700
	H	4.001272	6.128614	11.193123
	B	4.060102	8.430243	9.613162
	H	3.016292	8.990742	9.725800
	B	5.663212	9.159769	9.513089
	H	6.145602	10.241936	9.596050
	B	4.931829	8.461862	11.105751
	H	4.531767	8.944625	12.111752
	H	6.210890	8.637974	11.230881
$[B_{10}H_8O_2CCH_3*H^{fac}]^0$ TS2				
	O	7.276852	6.737342	11.195293
	O	6.573586	5.144937	9.812980
	C	7.329189	5.527875	10.769402
	C	8.254258	4.562466	11.412150
	H	7.702818	4.030462	12.194010
	H	9.088615	5.091169	11.871454
	H	8.601076	3.834437	10.678957
	B	5.580888	6.826580	7.707907
	H	5.807602	6.313185	6.662927
	B	5.756592	6.297897	9.275620
	B	4.149488	6.888493	8.654327
	H	3.153293	6.343183	8.300547
	B	5.022391	8.389743	8.060247
	H	4.751938	9.211532	7.245522
	B	6.588323	7.794447	8.701837
	H	7.740467	7.973081	8.445752

	B	6.195879	7.488702	10.476489
	B	4.532773	6.897775	10.464116
	H	3.941923	6.117289	11.143266
	B	3.885562	8.334217	9.682487
	H	2.843523	8.907607	9.694295
	B	5.914674	9.063790	9.719987
	H	6.369560	10.162510	9.736379
	B	4.956208	8.401449	11.117560
	H	4.750750	8.921910	12.161552
	H	4.640641	9.407214	9.436447
$[B_{10}H_8O_2CCH_3{*}H^{fac}]^0$ Iso 3				
	O	7.283442	6.648884	11.205067
	O	6.599333	5.083567	9.791195
	C	7.339715	5.447051	10.769677
	C	8.242844	4.464924	11.418149
	H	7.673768	3.936276	12.189634
	H	9.077837	4.979683	11.891917
	H	8.590143	3.736345	10.685824
	B	5.546383	6.758267	7.714917
	H	5.791632	6.320040	6.640555
	B	5.779574	6.238100	9.286171
	B	4.163046	6.715559	8.676750
	H	3.137217	6.225338	8.328528
	B	5.043928	8.350622	8.064978
	H	4.912647	9.016288	7.080095
	B	6.587678	7.745370	8.724846
	H	7.713032	7.944227	8.382112
	B	6.238992	7.435382	10.462118
	B	4.533805	6.860977	10.424448

	H	3.954461	6.095370	11.133565
	B	4.007449	8.407585	9.704325
	H	2.966849	8.960689	9.907410
	B	5.860929	9.032366	9.740340
	H	6.295415	10.138191	9.695488
	B	5.030016	8.416089	11.070562
	H	4.773356	8.866537	12.137210
	H	3.969858	8.958673	8.512888
$[B_{10}H_8O_2CCH_3{*}H^{fac}]^0$ Iso 4				
	O	7.299103	6.656462	11.195861
	O	6.558459	5.086890	9.811360
	C	7.329801	5.450337	10.764894
	C	8.242145	4.463987	11.393827
	H	7.682877	3.926778	12.166536
	H	9.081935	4.975996	11.861868
	H	8.581536	3.742446	10.650755
	B	5.592349	6.779041	7.729735
	H	5.794686	6.310973	6.659750
	B	5.763084	6.260228	9.301620
	B	4.164975	6.848755	8.677134
	H	3.165207	6.319580	8.310775
	B	5.094848	8.364811	8.031565
	H	4.915617	9.067314	7.085297
	B	6.633671	7.733571	8.725216
	H	7.784982	7.884427	8.450253
	B	6.237612	7.446150	10.478475
	B	4.545877	6.889078	10.455658
	H	3.941266	6.108478	11.124614
	B	3.985020	8.399650	9.728795

	H	2.957648	8.997760	9.749114
	B	5.890922	9.047137	9.742547
	H	6.324386	10.152961	9.728578
	B	5.026723	8.418228	11.093397
	H	4.833839	8.899786	12.160544
	H	4.637050	9.281995	8.847434
$[B_{10}H_8O_2CCH_3{*}H^{fac}]^0$ Iso 5				
	O	7.312634	6.668383	11.184311
	O	6.585178	5.093544	9.799673
	C	7.341547	5.460108	10.765418
	C	8.237273	4.471995	11.415015
	H	7.662936	3.948414	12.186068
	H	9.075843	4.980886	11.888677
	H	8.578982	3.739568	10.683823
	B	5.575323	6.769347	7.701568
	H	5.791034	6.262813	6.650538
	B	5.758477	6.242934	9.282800
	B	4.164336	6.828858	8.696882
	H	3.144407	6.342091	8.326466
	B	4.954420	8.328560	8.048640
	H	4.743027	9.110270	7.177668
	B	6.675861	7.652794	8.681724
	H	7.819025	7.807296	8.385819
	B	6.248181	7.454789	10.462107
	B	4.543819	6.855600	10.446575
	H	4.017611	6.048931	11.151581
	B	4.054465	8.400388	9.647792
	H	3.018066	8.985356	9.653788
	B	5.791300	9.064785	9.756195

	H	6.215566	10.172686	9.859565
	B	4.982843	8.360212	11.086913
	H	4.827772	8.897277	12.132320
	H	6.214865	8.981929	8.488083
$[B_{10}H_8O_2CCH_3*H^{fac}]^0$ Iso 6				
	O	7.299517	6.606410	11.250140
	O	6.596114	5.066921	9.810701
	C	7.336221	5.401159	10.790067
	C	8.228232	4.407231	11.433273
	H	7.668965	3.924614	12.241411
	H	9.095899	4.905569	11.864680
	H	8.522737	3.646108	10.711526
	B	5.562060	6.762091	7.710801
	H	5.837554	6.257155	6.674274
	B	5.799492	6.251696	9.295924
	B	4.182095	6.821041	8.709401
	H	3.207102	6.247349	8.340843
	B	5.041888	8.339294	8.102945
	H	4.793380	9.100314	7.221779
	B	6.666247	7.700889	8.626061
	H	7.782549	7.774346	8.206733
	B	6.251067	7.399812	10.557362
	B	4.546522	6.859382	10.460009
	H	4.067564	6.025833	11.168442
	B	4.059130	8.363766	9.649760
	H	3.046383	8.988623	9.619316
	B	5.706651	9.107171	9.640299
	H	6.163935	10.204131	9.669880
	B	5.014024	8.392542	11.078161

	H	4.815737	8.902846	12.131683
	H	6.986742	8.431301	9.671096

Optimized equilibrium model structures in dichloromethane

Compound	Atom	x	y	z
$[B_{10}H_8O_2CCH_3]^-$				
	O	7.313763	6.697223	11.197642
	O	6.597125	5.116389	9.813154
	C	7.357112	5.487351	10.776697
	C	8.260600	4.510506	11.422915
	H	7.694735	4.002183	12.211181
	H	9.109086	5.022453	11.876033
	H	8.592877	3.766594	10.698776
	B	5.591203	6.837329	7.726907
	H	5.797277	6.339362	6.660329
	B	5.783547	6.280814	9.290338
	B	4.172721	6.869713	8.695957
	H	3.172546	6.324035	8.321836
	B	5.030368	8.394869	8.145063
	H	4.768789	9.170680	7.266376
	B	6.615380	7.773679	8.723033
	H	7.767606	7.958022	8.439991
	B	6.241220	7.480509	10.470859
	B	4.544965	6.882771	10.467419
	H	4.014725	6.059661	11.162834
	B	4.042257	8.413704	9.670827
	H	2.989322	8.990353	9.681598
	B	5.764422	9.048211	9.688952

	H	6.218719	10.157908	9.702272
	B	4.996849	8.410176	11.086083
	H	4.805858	8.891947	12.162936
$[B_{10}H_8O_2CCH_3{*}H^{fac}]^0$ Iso 1				
	O	7.284547	6.733686	11.186792
	O	6.590200	5.135557	9.812347
	C	7.337641	5.512141	10.785960
	C	8.223605	4.546098	11.460551
	H	7.629616	4.032933	12.225274
	H	9.050860	5.063708	11.944464
	H	8.583725	3.805145	10.746737
	B	5.580674	6.851546	7.693494
	H	5.780072	6.408715	6.608948
	B	5.793425	6.278504	9.265157
	B	4.180012	6.810260	8.661243
	H	3.181605	6.278184	8.285901
	B	4.983856	8.395907	8.156641
	H	4.720977	9.166773	7.283355
	B	6.613769	7.747234	8.689745
	H	7.742998	7.994195	8.394783
	B	6.258420	7.497459	10.420621
	B	4.535805	6.885243	10.419536
	H	4.060679	6.112364	11.193291
	B	3.936764	8.330049	9.629414
	H	2.912980	8.933723	9.653239
	B	5.843410	9.066371	9.672658
	H	6.314414	10.155910	9.592647
	B	4.997602	8.385534	11.116568
	H	4.848999	8.687134	12.253433

	H	4.767740	9.490889	10.492966
$[B_{10}H_8O_2CCH_3{*}H^{fac}]^0$ TS1				
	O	7.218734	6.664433	11.253439
	O	6.625316	5.107468	9.789743
	C	7.334059	5.462085	10.795782
	C	8.274609	4.525732	11.435150
	H	8.045856	4.469743	12.502567
	H	9.287346	4.925647	11.327770
	H	8.206300	3.542065	10.974814
	B	5.672825	6.844017	7.711820
	H	5.983237	6.396080	6.655657
	B	5.803569	6.248977	9.260197
	B	4.197943	6.821392	8.616297
	H	3.222622	6.249699	8.237997
	B	4.990516	8.348617	8.084085
	H	4.784170	9.132199	7.208373
	B	6.614236	7.788610	8.798059
	H	7.758909	8.009690	8.538366
	B	6.198012	7.404008	10.487082
	B	4.545181	6.845146	10.399812
	H	3.994879	6.088102	11.137901
	B	3.974229	8.352120	9.561038
	H	2.899370	8.867684	9.571276
	B	5.633338	9.114151	9.628988
	H	6.103137	10.203143	9.506828
	B	4.843295	8.405163	11.060804
	H	4.443320	8.742686	12.125761
	H	5.889694	9.152522	10.989709
$[B_{10}H_8O_2CCH_3{*}H^{fac}]^0$ Iso 2				

	O	7.302128	6.668642	11.215339
	O	6.573631	5.122910	9.809620
	C	7.352635	5.460271	10.765529
	C	8.274241	4.484995	11.371796
	H	7.738210	3.991545	12.190593
	H	9.145908	4.994142	11.781857
	H	8.561769	3.732781	10.637863
	B	5.632842	6.820633	7.749408
	H	5.911476	6.284585	6.725771
	B	5.762521	6.274374	9.313330
	B	4.188351	6.844238	8.707189
	H	3.186909	6.287826	8.378341
	B	5.013984	8.358965	8.072389
	H	4.775346	9.104084	7.170585
	B	6.605799	7.824925	8.751663
	H	7.752491	7.998324	8.464171
	B	6.253432	7.465244	10.539549
	B	4.557970	6.897731	10.468918
	H	3.994035	6.131258	11.188064
	B	4.064496	8.428243	9.615995
	H	3.018096	8.988769	9.722909
	B	5.668459	9.154620	9.518067
	H	6.137241	10.243203	9.593800
	B	4.939499	8.459473	11.104570
	H	4.537628	8.949637	12.105819
	H	6.217143	8.636981	11.235077
$[B_{10}H_8O_2CCH_3*H^{fac}]^0$ TS2				
	O	7.277808	6.738907	11.190421
	O	6.576827	5.148476	9.808249

	C	7.338422	5.522850	10.771150
	C	8.252675	4.561706	11.413178
	H	7.686241	4.037396	12.191161
	H	9.087316	5.085157	11.877909
	H	8.600215	3.829696	10.684164
	B	5.582272	6.826170	7.710684
	H	5.805580	6.314896	6.661627
	B	5.766365	6.292295	9.277041
	B	4.154796	6.885594	8.658491
	H	3.155011	6.342424	8.305549
	B	5.019605	8.388336	8.063057
	H	4.745200	9.206881	7.244979
	B	6.586967	7.795679	8.699662
	H	7.736981	7.982452	8.439867
	B	6.203472	7.483717	10.477479
	B	4.535323	6.897474	10.463070
	H	3.937842	6.118115	11.137618
	B	3.895194	8.340123	9.680614
	H	2.849358	8.905479	9.698934
	B	5.907890	9.062773	9.718656
	H	6.358245	10.162182	9.739048
	B	4.961287	8.397697	11.115170
	H	4.753654	8.919855	12.158782
	H	4.634322	9.422963	9.434950
$[B_{10}H_8O_2CCH_3{*}H^{fac}]^0$ Iso 3				
	O	7.282372	6.649508	11.204427
	O	6.601900	5.086383	9.789555
	C	7.348458	5.441232	10.773349
	C	8.244433	4.464678	11.418041

	H	7.660449	3.932639	12.177586
	H	9.073701	4.976141	11.905155
	H	8.601153	3.741022	10.685093
	B	5.546660	6.759988	7.719741
	H	5.789265	6.321140	6.643061
	B	5.786683	6.232180	9.290393
	B	4.165806	6.716219	8.681011
	H	3.139844	6.222505	8.333701
	B	5.036562	8.350552	8.072633
	H	4.896552	9.018824	7.090865
	B	6.586489	7.744629	8.724535
	H	7.710278	7.947979	8.377457
	B	6.245143	7.429004	10.465922
	B	4.535771	6.861439	10.425335
	H	3.952590	6.098616	11.134922
	B	4.012603	8.408094	9.696279
	H	2.972160	8.964695	9.889771
	B	5.861017	9.028404	9.738524
	H	6.297880	10.134701	9.694564
	B	5.031200	8.412808	11.066474
	H	4.774233	8.866388	12.133728
	H	3.960147	8.972634	8.506648
$[B_{10}H_8O_2CCH_3{*}H^{fac}]^0$ Iso 4				
	O	7.297108	6.655050	11.196090
	O	6.560714	5.090497	9.807792
	C	7.337787	5.442853	10.767707
	C	8.241704	4.460978	11.392934
	H	7.669146	3.927918	12.160409
	H	9.081090	4.967704	11.867377

	H	8.582926	3.738628	10.651350
	B	5.595131	6.781805	7.733960
	H	5.800489	6.315449	6.662310
	B	5.771144	6.256151	9.306544
	B	4.171862	6.843147	8.681063
	H	3.168076	6.316170	8.316848
	B	5.085920	8.365214	8.040886
	H	4.906313	9.060719	7.089097
	B	6.631203	7.738475	8.727685
	H	7.779738	7.894126	8.443178
	B	6.246068	7.440120	10.484553
	B	4.548149	6.890274	10.454945
	H	3.937683	6.113633	11.123687
	B	3.995955	8.405157	9.720905
	H	2.962939	8.994057	9.753370
	B	5.894481	9.047112	9.745306
	H	6.327850	10.153041	9.726063
	B	5.032420	8.417158	11.089760
	H	4.828209	8.900715	12.155955
	H	4.611513	9.296670	8.825830
$[B_{10}H_8O_2CCH_3{*}H^{fac}]^0$ Iso 5				
	O	7.305821	6.668289	11.186799
	O	6.585041	5.097110	9.798089
	C	7.348146	5.455136	10.769083
	C	8.240503	4.473338	11.410816
	H	7.652624	3.939839	12.166398
	H	9.070675	4.979874	11.901246
	H	8.595260	3.751038	10.675466
	B	5.574774	6.769371	7.708326

	H	5.786603	6.260278	6.655798
	B	5.763944	6.237135	9.289162
	B	4.165724	6.827322	8.700738
	H	3.143824	6.341162	8.328080
	B	4.958324	8.328360	8.051411
	H	4.736496	9.107964	7.180958
	B	6.674259	7.655138	8.685087
	H	7.815318	7.810275	8.387710
	B	6.250927	7.447532	10.468082
	B	4.541145	6.857423	10.448915
	H	4.003915	6.056141	11.152004
	B	4.056923	8.400108	9.646059
	H	3.018165	8.985961	9.651588
	B	5.796340	9.061072	9.753244
	H	6.215844	10.170767	9.848746
	B	4.986612	8.361152	11.083250
	H	4.824515	8.900733	12.127942
	H	6.223069	8.987297	8.485014
$[B_{10}H_8O_2CCH_3{*}H^{fac}]^0$ Iso 6				
	O	7.285835	6.604258	11.256894
	O	6.592929	5.070512	9.809193
	C	7.340321	5.395406	10.794185
	C	8.232705	4.410428	11.426227
	H	7.668145	3.927183	12.231996
	H	9.098778	4.910010	11.859460
	H	8.529874	3.652119	10.702598
	B	5.569355	6.758984	7.720330
	H	5.838683	6.252913	6.681568
	B	5.801955	6.244540	9.303913

	B	4.183263	6.820378	8.712379
	H	3.205410	6.253792	8.334069
	B	5.041354	8.334866	8.104589
	H	4.784939	9.095555	7.222391
	B	6.660623	7.708891	8.634398
	H	7.773322	7.779850	8.209299
	B	6.249167	7.389592	10.569307
	B	4.542156	6.860196	10.460640
	H	4.047234	6.032945	11.165033
	B	4.063012	8.366307	9.646940
	H	3.047003	8.990843	9.621105
	B	5.723418	9.094207	9.641690
	H	6.163477	10.198286	9.665177
	B	5.016243	8.391991	11.074589
	H	4.806531	8.908725	12.124854
	H	7.012079	8.434820	9.663741

Optimized equilibrium model structures used for analysis of the mechanism for the protonation of $[B_{10}H_8O_2CCH_3]^-$

Compound	Atom	x	y	z
CF_3SO_3H				
	S	1.849032	2.796840	11.451578
	O	0.886440	2.931307	12.709077
	O	1.782444	4.041592	10.731160
	O	1.677709	1.511331	10.815756
	H	0.604992	2.055318	13.041152
	C	3.492373	2.756880	12.313184
	F	3.673316	3.878385	12.998016

	F	3.530683	1.717889	13.141650
	F	4.446525	2.632799	11.397750
**Comp**				
	O	7.397099	6.884796	11.074299
	O	6.209599	5.191881	10.264510
	C	7.086522	5.640430	11.085498
	C	7.713565	4.730121	12.067522
	H	7.089345	4.731098	12.967963
	H	8.708220	5.087782	12.332621
	H	7.753403	3.715858	11.670702
	B	5.619984	6.315706	7.673865
	H	5.627017	5.453970	6.847404
	B	5.727160	6.286539	9.339281
	B	4.313772	7.059092	8.506226
	H	3.183406	6.727359	8.287795
	B	5.539034	8.011183	7.512312
	H	5.459155	8.491588	6.416676
	B	6.911529	7.210900	8.347649
	H	8.052332	6.959431	8.074188
	B	6.554202	7.638447	10.071632
	B	4.760373	7.566902	10.183211
	H	4.051471	7.221857	11.086462
	B	4.678389	8.810014	8.893325
	H	3.821778	9.606665	8.598299
	B	6.499598	8.919207	8.781599
	H	7.236141	9.800519	8.442938
	B	5.653952	9.027540	10.272836
	H	5.656177	9.875701	11.112544
	S	2.493916	12.048007	10.206707

	O	2.888071	10.621833	10.742105
	O	1.566660	12.620588	11.152089
	O	2.227175	12.025494	8.786090
	H	3.407807	10.100912	10.070004
	C	4.100665	12.951137	10.417699
	F	4.497398	12.892876	11.684521
	F	5.027207	12.405998	9.637509
	F	3.920640	14.223313	10.069357
**TS**				
	O	6.573567	6.615451	11.428391
	O	5.835959	5.134041	9.947339
	C	6.419483	5.394218	11.060089
	C	6.943156	4.298125	11.899007
	H	7.988096	4.133790	11.613180
	H	6.381439	3.382354	11.718017
	H	6.910945	4.580761	12.951461
	B	5.789180	6.887030	7.632655
	H	6.110934	6.320401	6.635752
	B	5.486045	6.399209	9.212667
	B	4.241904	7.276764	8.237530
	H	3.251436	6.972715	7.642076
	B	5.528302	8.560465	7.913097
	H	5.673583	9.308711	6.990424
	B	6.759212	7.588265	8.835704
	H	7.952808	7.506565	8.817711
	B	5.958655	7.551820	10.430983
	B	4.196129	7.368938	10.031503
	H	3.364468	6.779181	10.655478
	B	4.224714	8.880813	9.119731

	H	3.381820	9.677770	8.857718
	B	6.098365	9.114915	9.577169
	H	6.843710	10.042462	9.610824
	B	4.851403	8.779278	10.758426
	H	4.530205	9.191901	11.825166
	S	3.672662	12.336346	10.649702
	O	4.922893	11.556977	10.352547
	O	3.909558	13.451290	11.560883
	O	2.496026	11.507637	10.885598
	H	4.963914	10.159459	10.231828
	C	3.354956	13.153677	9.018121
	F	4.385557	13.933124	8.669515
	F	3.168940	12.248424	8.053158
	F	2.257890	13.918951	9.092881

Optimized equilibrium model structures for supramolecular associates of $CF_3SO_3^-$

Compound	Atom	x	y	z
$CF_3SO_3^-$				
	S	1.771385	2.812592	11.530485
	O	0.876202	2.919248	12.691528
	O	1.842901	4.010811	10.682043
	O	1.707991	1.535077	10.805980
	C	3.453186	2.758078	12.308937
	F	3.699598	3.872255	13.017556
	F	3.571173	1.711969	13.142809
	F	4.416086	2.646992	11.378833

Compound	Atom	x	y	z

$CF_3SO_3^-$_H_2O				
	S	1.297583	4.820432	12.186725
	O	1.857764	3.687278	12.936505
	O	0.235108	4.447542	11.230328
	O	2.266214	5.800727	11.687927
	C	0.362681	5.759760	13.480772
	F	-0.592180	4.996579	14.032643
	F	1.179130	6.170904	14.462884
	F	-0.228878	6.844202	12.957703
	O	-0.315412	1.699884	11.849410
	H	0.421147	1.646657	12.469471
	H	-0.266819	2.618950	11.537357

Compound	Atom	x	y	z
$CF_3SO_3^-$_H_3O				
	S	1.232304	3.140503	11.872670
	O	0.888656	4.315136	12.837227
	O	1.438424	3.666676	10.544286
	O	0.371562	1.999841	12.115500
	C	2.903971	2.656137	12.509390
	F	3.753298	3.673194	12.401285
	F	2.805713	2.302226	13.791013
	F	3.359045	1.625051	11.802926
	O	-0.297732	3.572434	14.930755
	H	0.174457	3.722836	15.761780
	H	-0.619297	2.660045	14.952840
	H	0.406089	3.992990	13.693686

Compound	Atom	x	y	z

$CF_3SO_3^-$_CF_3SO_3H				
	S	1.337071	3.010722	11.702255
	O	0.744906	3.483374	12.994035
	O	1.716548	4.114842	10.825511
	O	0.637162	1.880708	11.102692
	C	2.955808	2.310249	12.268482
	F	3.713993	3.259950	12.831578
	F	2.768340	1.338637	13.165833
	F	3.630013	1.799875	11.229211
	O	-0.047577	0.111003	13.949303
	O	-1.473936	0.502294	15.988297
	S	-1.022079	0.899811	14.671709
	O	-0.654467	2.404275	14.704516
	H	-0.051321	2.754761	13.913852
	C	-2.541692	0.938873	13.611461
	F	-3.496081	1.659971	14.199528
	F	-2.262640	1.472305	12.426080
	F	-2.985540	-0.307000	13.437974
